# Using computational models to predict *in vivo* synaptic inputs to interneuron specific 3 (IS3) cells of CA1 hippocampus that also allow their recruitment during rhythmic states

**DOI:** 10.1371/journal.pone.0209429

**Published:** 2019-01-08

**Authors:** Alexandre Guet-McCreight, Frances K. Skinner

**Affiliations:** 1 Krembil Research Institute, University Health Network, Toronto, Ontario, Canada; 2 Department of Physiology, University of Toronto, Toronto, Ontario, Canada; 3 Departments of Medicine (Neurology) and Physiology, University of Toronto, Toronto, Ontario, Canada; Georgia State University, UNITED STATES

## Abstract

Brain coding strategies are enabled by the balance of synaptic inputs that individual neurons receive as determined by the networks in which they reside. Inhibitory cell types contribute to brain function in distinct ways but recording from specific, inhibitory cell types during behaviour to determine their contributions is highly challenging. In particular, the *in vivo* activities of vasoactive intestinal peptide-expressing interneuron specific 3 (IS3) cells in the hippocampus that only target other inhibitory cells are unknown at present. We perform a massive, computational exploration of possible synaptic inputs to IS3 cells using multi-compartment models and optimized synaptic parameters. We find that asynchronous, *in vivo*-like states that are sensitive to additional theta-timed inputs (8 Hz) exist when excitatory and inhibitory synaptic conductances are approximately equally balanced and with low numbers of activated synapses receiving correlated inputs. Specifically, under these balanced conditions, the input resistance is larger with higher mean spike firing rates relative to other activated synaptic conditions investigated. Incoming theta-timed inputs result in strongly increased spectral power relative to baseline. Thus, using a generally applicable computational approach we predict the existence and features of background, balanced states in hippocampal circuits.

## Introduction

A dizzying array of morphological, molecular, and electrophysiological details for different cell types exist and appropriate classifications are being determined [1]. How these different cell types contribute to brain function is challenging to determine, but it is clear that a homeostatic balance of cell excitability, together with excitatory and inhibitory synaptic inputs is essential for normal brain function [2–5]. The irregular firing of neurons *in vivo* is well-known and is believed to confer computational benefits, with inhibition being recognized as a crucial shaper of these asynchronous activities [6, 7]. Recently, in directly fitting a deterministic firing network model to several sets of *in vivo* multi-neuron data, it was found that the intrinsically generated variability obtained in experiment was mainly due to feedback inhibition [8]. In essence, it is critical to understand these inhibitory components. However, we are cognisant of the much more diverse nature of inhibitory cells relative to excitatory cells in our brains, despite their smaller overall numbers [9–11]. While the examination of individual neuron activities in the behaving animal is becoming less uncommon, there are certainly more caveats and technical difficulties relative to *in vitro* studies. Further, the smaller numbers and sizes of inhibitory cells as well as being in hard to access locations create additional challenges for identification and patching. Indeed, the activity of several inhibitory cell types *in vivo* remains unknown.

One such cell type that suffers from these difficulties are hippocampal CA1 interneuron specific type 3 (IS3) interneurons. IS3 cells are a vasoactive intestinal polypeptide-positive (VIP+) and calretinin-positive (CR+) cell type with cell bodies found in the stratum radiatum and stratum pyramidale of the CA1 [12–15], an area in CA1 more predominantly populated by pyramidal cells as well as some parvalbumin-positive (PV+) cell types. Compared to pyramidal cells, which make up approximately 80-90% of neurons in CA1 [16, 17], IS3 cells make up less than one percent of the CA1 neurons [18], making them much more difficult to locate. Previous work has circumvented these issues through usage of GFP-VIP mouse lines such that IS3 cells can be readily identified *in vitro* [19]. Further, new calcium imaging techniques show promise in recording network activity of IS3 cells *in vivo* [20], but electrophysiological recordings during *in vivo* hippocampal rhythms such as theta has not been characterized. *In vivo* juxtacellular recordings have been obtained from other hippocampal inhibitory cell types such as oriens-lacunosum/moleculare (OLM) cells, ivy cells, bistratified cells, axo-axonic cells, and basket cells [21–25], yet, due to the difficult nature of these techniques, these experiments often suffer from low numbers of recordings. Although IS3 cells only represent a small percentage of CA1 network neurons, understanding their contributions to network activity is a compelling exploration in hippocampal research due to their distinct connections exclusively onto other inhibitory interneurons, such as OLM and bistratified cells [19, 26]. This unique circuitry suggests a potential function in the disinhibition of pyramidal cells. Such a contribution to network function has in fact been observed in VIP+ cells of different cortical areas during a variety of different behavioral contexts [27–34].

Despite the challenges of *in vivo* recordings, much can be said about the *in vivo* state. That is, *in vivo* recordings are irregular and asynchronous and constitute “high-conductance” states [35], where a high level of synaptic bombardment causes the cells’ subthreshold membrane potential to be more depolarized, to have larger fluctuations, and to have smaller input resistances [36]. To move toward an understanding of IS3 cell contributions to brain function, we use a computational approach by necessity, as recording from these cell types *in vivo* is not possible at the present time. We explore what excitatory and inhibitory inputs could bring about asynchronous states in models of IS3 cells and also be sensitive to theta frequency inputs as has been shown *in vitro* [19]. We use two variant IS3 cell models from our previous work [37] and find that excitatory and inhibitory synaptic conductances need to be approximately, equally balanced with correlated inputs (common inputs). As well, we observed differences depending on which IS3 cell model variant was used. This work contributes to an understanding of excitatory and inhibitory balances in hippocampal circuits.

## Results

**Fig 1:**
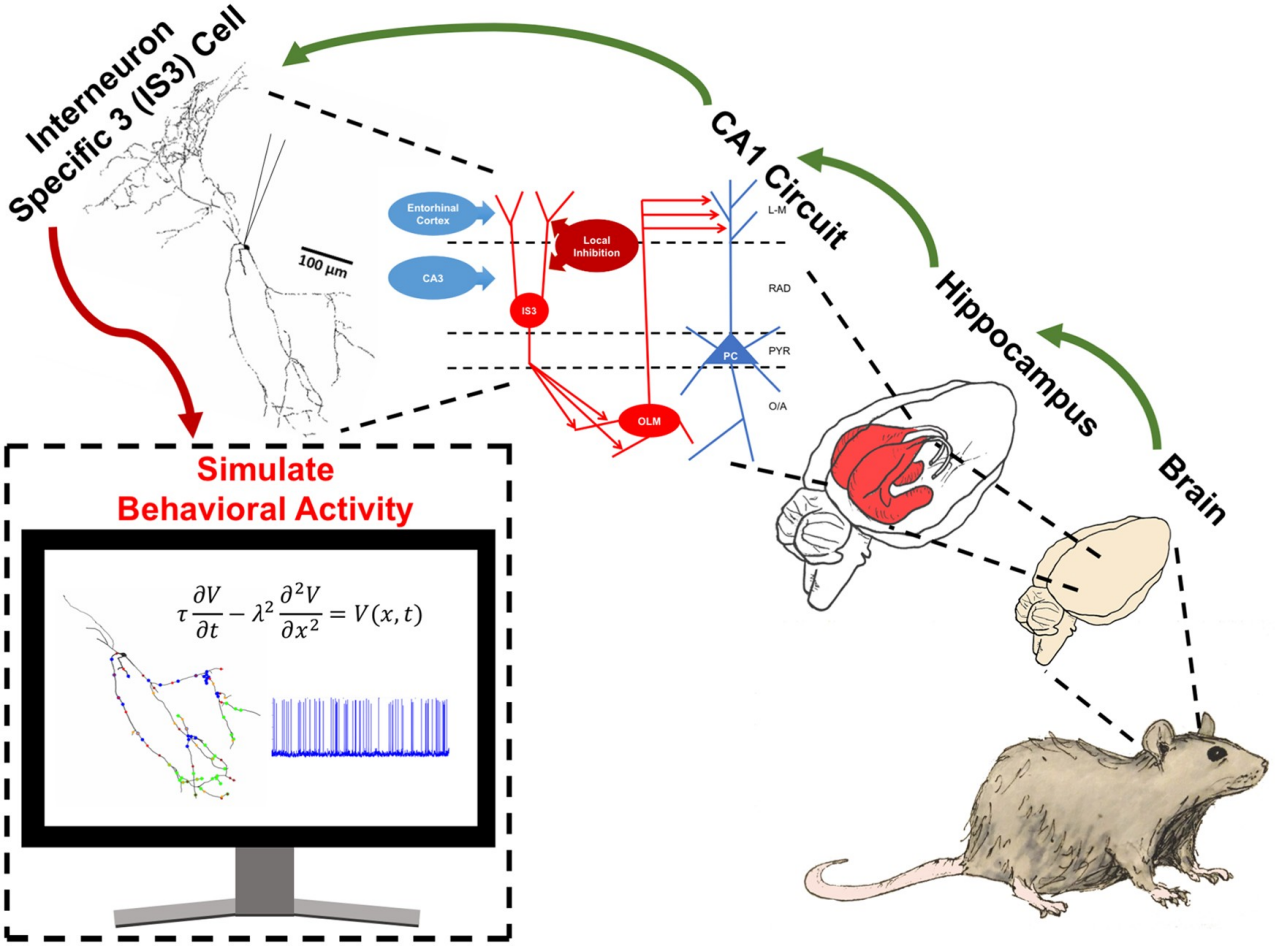
Schematic illustrating computational approach and microcircuit schematic with IS3 cells. In vivo behavioural activity of IS3 cells is determined using data-driven mathematical models to simulate *in vivo*-like states (red arrow). As schematized, our computational approach considers ***IS3 cells*** embedded in ***CA1 circuits*** of the ***hippocampus*** of the ***brain*** of a mouse (green arrows). Acronyms in CA1 circuit: CA3—Cornu Ammonis area 3; IS3—Interneuron Specific type 3 cell; OLM—Oriens Lacunosum Moleculare cell; PC—Pyramidal cell; L-M—stratum lacunosum moleculare; RAD—stratum radiatum; PYR—stratum pyramidale; O/A—stratum oriens/alveus. The “mouse”, “brain”, and “hippocampus” images were provided with permission from A. Sherrington under a CC-BY 4.0 license.

Due to the diversity and details of inhibitory cells it is challenging to understand their roles and contributions in brain circuits and behaviours. We used a computational approach to simulate *in vivo*-like activities so as to determine how much and what balance of excitatory and inhibitory synaptic inputs inhibitory cell subtypes might receive in the behaving animal. We illustrate this overall approach in Fig 1 for IS3 cells that we examine here.

We used our previously developed multi-compartment models of IS3 cells in which the electrophysiological features of IS3 cells were captured [37]. Our models are detailed in terms of morphology and the inclusion of four types of ion channels, and we used two variant IS3 cell models. One model variant has A-type potassium channels in its soma and proximal dendrites, and the other has A-type potassium channels restricted to its soma. In our previous work [37] we referred to these model variants as SDprox1 and SDprox2 respectively. Here, we will refer to these models as “AType+” and “AType-” to reflect in a more straightforward fashion which model variant has A-type potassium channels in their dendrites (AType+) or not (AType-). We included both model variants as we had found that either model variant could capture the electrophysiology of IS3 cells [37]. In addition, given the known effects of dendritic A-type potassium channels on synaptic integration [38] it is possible that the presence or absence of dendritic A-type potassium channels in our models would generate differences in our results. Further model details and parameter values are given in the Methods section.

To perform parameter explorations of input regimes that yield *in vivo*-like states, we first considered the types of layer-specific excitatory and inhibitory inputs to IS3 cells. Within the hippocampus, IS3 cells have dendrites that extend into the stratum radiatum and stratum pyramidale, which allow them to receive excitatory inputs from both CA3 and entorhinal cortex. Layer-specific inhibitory inputs to IS3 cells are not characterized, though there are several possible inhibitory presynaptic populations to IS3 cells. For this reason we considered one generic population of inhibitory inputs, one population of proximal excitatory inputs, and one population of distal excitatory inputs. This is illustrated in the ‘CA1 circuit’ schematic of Fig 1. Using our two IS3 cell multi-compartment models, we investigated a wide range of parameter values characterizing excitatory and inhibitory synaptic inputs onto these cell models. We explored the full range of possible input parameter combinations based on what is known experimentally to obtain *in vivo*-like states, and both common or independent excitatory and inhibitory inputs were considered. This is illustrated in S1A–S1C Fig, and the details of our parameter exploration strategy and setup are given in the Methods.

We devised an *in vivo*-like (IVL) metric (Eq 5) to identify IVL states. This IVL metric was based on three criteria: the level of depolarization, the amount of fluctuations as given by the membrane potential standard deviation, and the interspike interval coefficient of variation. Thresholds for the criteria were obtained from the literature and customized according to the properties of the IS3 cell model where possible. As detailed in the Methods, output from our IS3 cell models were assumed to sufficiently represent an IVL state if our IVL metric had a value of three (i.e. satisfied all three criteria listed above). We subtracted a value of four from the IVL metric if the average spike amplitude was below a certain threshold value so that a negative IVL metric would reflect that the output was nearing depolarization block (DB). Scenarios with an IVL metric of zero (i.e. did not satisfy any of the criteria) were labeled as being in non-*in vivo*-like (NIVL) states. Consideration of these additional DB and NIVL states helped us better interpret our results.

### Common inputs and an inhibitory dominance promote *in vivo*-like (IVL) states in IS3 cells

From a full set of parameter explorations, we display our results using clutter-based dimensional reordering (CBDR) plots [39]. In this visualization plotting technique, multiple parameter ranges are re-stacked at different corresponding resolutions along the same x- or y- axes. This enables one to plot multi-dimensional parameter sets as two-dimensional (2D) plots and easily observe which parameter sets give rise to *in vivo*-like states. A CBDR plot is illustrated in S1D Fig together with an example voltage output. From the scale bars of the CBDR plot, one can extract the unique combination of parameters (i.e. excitatory and inhibitory spike rates and numbers of synapses) for each pixel in the 2D plot.

CBDR plots for our full sets of explorations using the AType+ and AType- models and with common or independent inputs are shown in S2 Fig. From these plots we observed that common excitatory inputs promote IVL states, and having common inputs for both excitatory and inhibitory synapses maximized the number of IVL states. We also observed that when comparing the AType+ and AType- models that the AType- model enters DB much more readily than the AType+ model. This is likely due to the lack of dendritic A-type potassium in the AType- model making it more excitable. Further details are given in S2 Fig and its caption.

Moving forward, we focused only on scenarios in which there were common inputs since it is clear that having common inputs maximized the space of parameter sets that could generate IVL states. However, we retained our consideration of both AType+ and AType- models as differences seem to be apparent. To get a handle on our many outputs, we examined the balance of excitation and inhibition for IVL, NIVL and DB states from our parameter explorations. We used two different excitation-inhibition (EI) metrics as given by Eqs 6 and 7 in the Methods. Briefly, EI metric #1 computes the difference between normalized excitatory and inhibitory input spike rates and numbers of synapses. EI metric #2 computes the cumulative difference in excitatory and inhibitory spike rates across all synapses. For both metrics, a negative value implies a dominance of inhibitory inputs. A metric value of zero implies that excitation and inhibition are approximately equal. We note that since synaptic inputs are not present at the same location and our metrics do not take spatial aspects into consideration, an EI metric value of zero does not necessarily mean that inputs are perfectly balanced in terms of amount and spatial spread. Histogram distributions of our EI metrics for the IVL, NIVL, and DB states are shown in plots in S3 Fig. From these plots, it is clear that the majority of IVL state scenarios exhibited an inhibitory dominance. Predictably, the DB distributions tended to lean toward excitatory-dominant regimes with low amounts of inhibition as represented by positive EI metric values. These plots also highlight the larger number of DB scenarios generated when using the AType- model rather than the AType+ model as already noted above. Looking closely at these plots suggests that subpopulations of IVL states with balanced excitatory and inhibitory inputs may be present. Further details are given in S3 Fig and its caption.

### Low amounts of balanced synaptic inputs and high amounts of inhibitory-dominant synaptic inputs can both generate *in vivo*-like (IVL) states in IS3 cells

To determine subspaces of parameter sets as well as uncover relationships between IVL states and balances between excitation and inhibition, we divided the parameter space into 16 pools by splitting each parameter into high and low ranges as specified in Table 1. The acronym naming scheme we used is given in Table 2 and these acronyms are used in subsequent figures.

**Table 1:** Ranges for each parameter when splitting the parameter space into 16 pools.

Parameter	Low	High
Excitatory Spike Rate (Hz)	0-15	20-30
Inhibitory Spike Rate (Hz)	0-50	60-100
Number of Excitatory Synapses	18-765	783-1530
Number of Inhibitory Synapses	4-172	176-344

**Table 2:** Naming scheme for the 16 different pool labels (see Fig 2).

Acronym	Pool Label Meaning
**LLLL**	**L** (Low) Number of Inhibitory Synapses;
	**L** Number of Excitatory Synapses;
	**L** Inhibitory Spike Rates;
	**L** Excitatory Spike Rates
**HHHH**	**H** (High) Number of Inhibitory Synapses;
	**H** Number of Excitatory Synapses;
	**H** Inhibitory Spike Rates;
	**H** Excitatory Spike Rates
**LLHH**	**L** Number of Inhibitory Synapses;
	**L** Number of Excitatory Synapses;
	**H** Inhibitory Spike Rates;
	**H** Excitatory Spike rates
**HHLL**	**H** Number of Inhibitory Synapses;
	**H** Number of Excitatory Synapses;
	**L** Inhibitory Spike Rates;
	**L** Excitatory Spike rates
etc.	**…**

**Fig 2:**
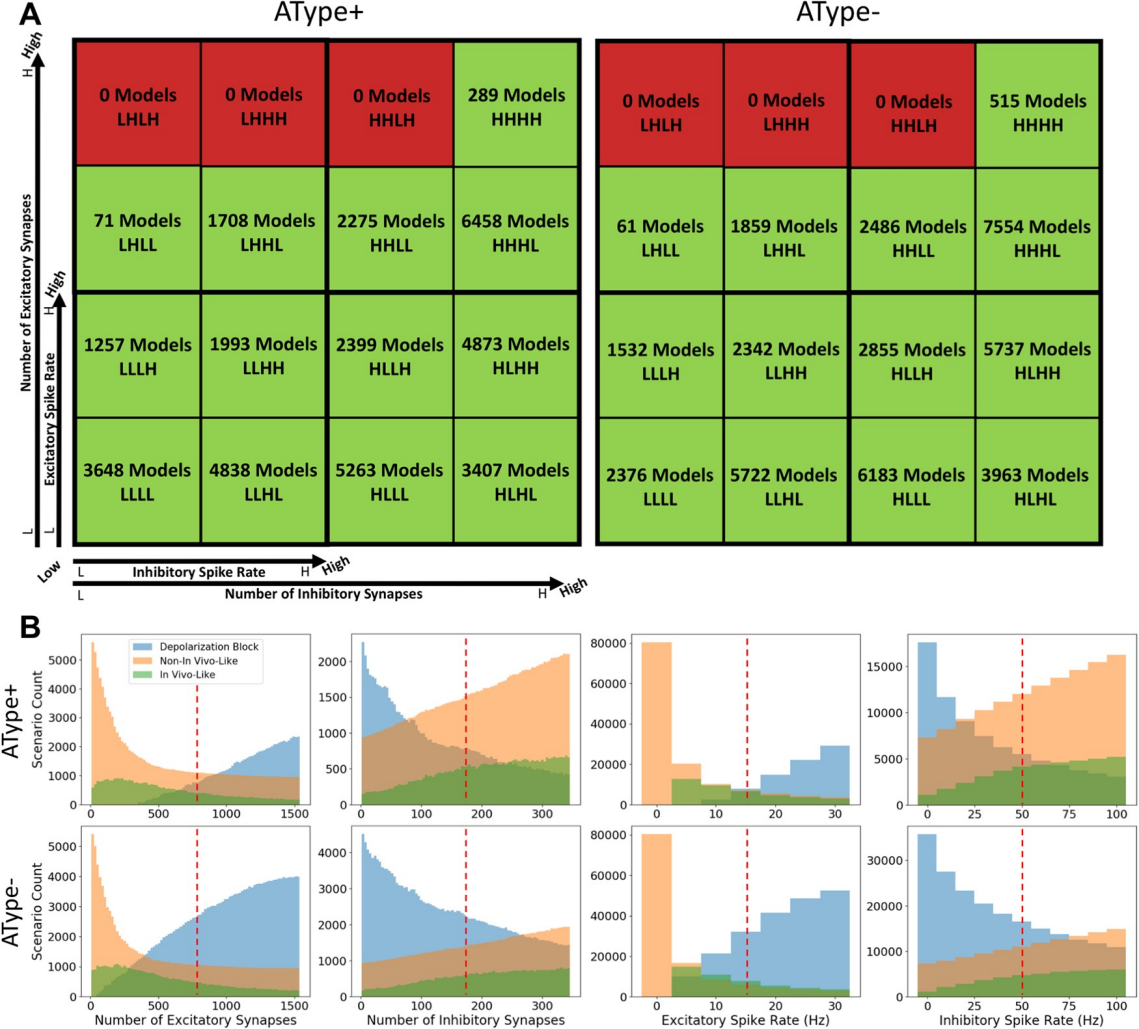
Dividing up the input parameter space. **A**: Schematic of parameter space pool divisions. Pools in green generated IVL states while pools in red did not generate any. Number of IVL scenarios for each pool are indicated in each square as well as the pool labels. As detailed in Table 2, labels L or H represent “low” or “high” parameter ranges for excitatory and inhibitory numbers and spike rates. The smaller axes refer to each quarter of the entire large square. Axis labels are the same for AType+ and AType-. **B**: Histogram distributions of input parameters for each of the defined states (i.e. IVL, NIVL, and DB) for the AType+ and AType- models. Red lines delineate the ranges of parameters when splitting into 16 pools as schematized in **A** (see Table 1). Note that IVL states tend to occur with larger amounts of inhibition. This can be plotted in the 16 pool scheme of **A** (see additional plot 6 on osf.io/6zg7a) where we can also observe that mean spike rates do not vary largely across pools, and further note that having a larger amount of inputs does not necessarily result in higher mean spike rates.

In Fig 2A we show these 16 pools as red or green squares where red squares represent parameter sets where no IVL states were found. For both the AType+ and AType- models, there were three pools where no IVL states were found and they encompassed parameter sets with high excitatory spike rates and high numbers of excitatory synapses. For the other 13 pools the number of IVL states is indicated along with the pool label acronym (see Table 2). In Fig 2B we plot histograms of IVL, NIVL and DB states, where the delineation of the high and low parameter values of the 16 pools is shown with a red dashed line. From these plots it is clear that pools with high amounts of inhibition and low amounts of excitation had large numbers of NIVL states, and there were large amounts of DB states for the inverse.

**Fig 3:**
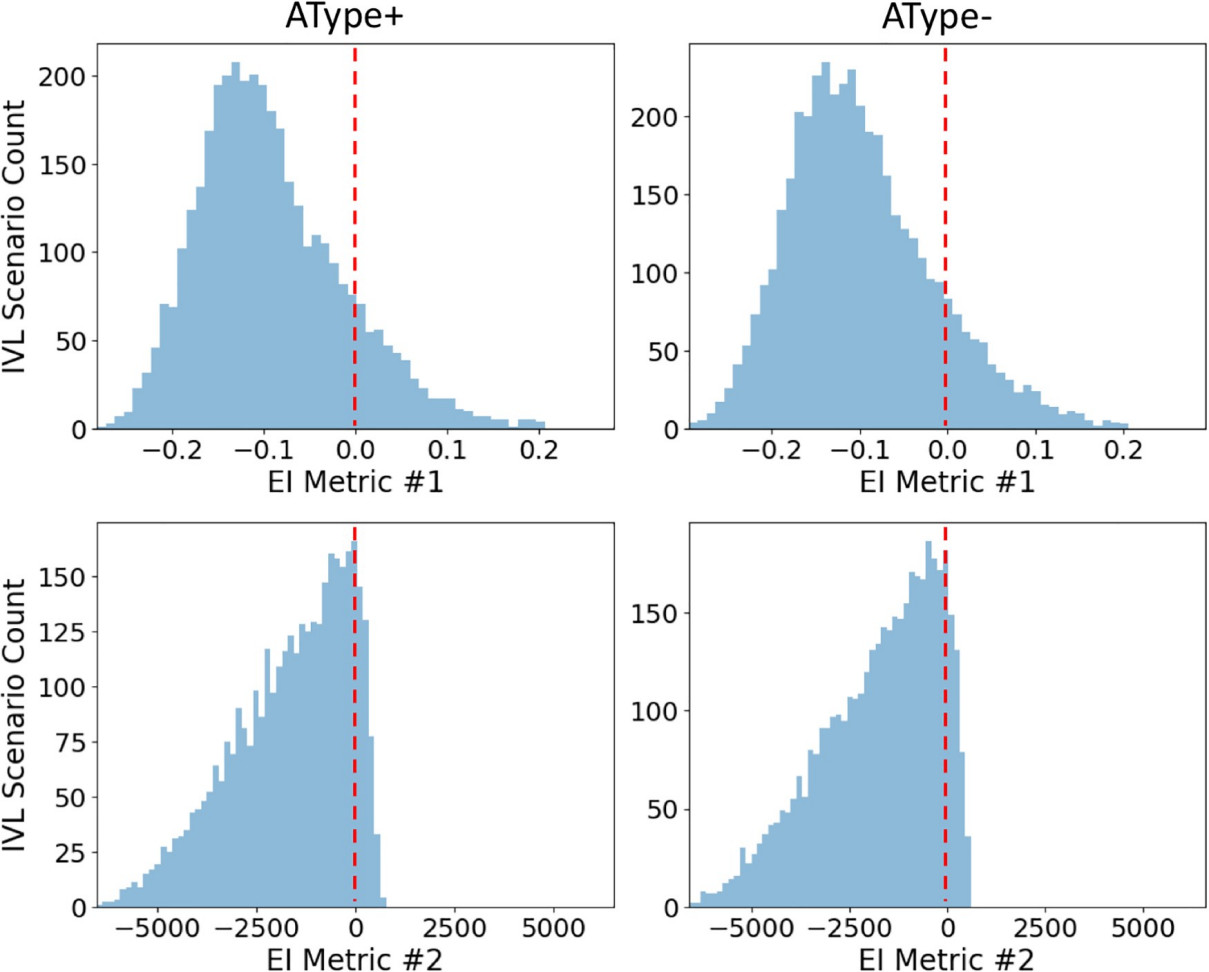
*In vivo*-like (IVL) EI metrics for one of the parameter pools. Distributions of EI metric values for IVL states for the LLLL pool for both the AType+ and AType- models. Red dashed lines indicate the EI value of zero (for both metrics) where excitation and inhibition are even.

EI metrics for the 16 pools are shown in S4 Fig (metric #1) and S5 Fig (metric #2) for IVL, NIVL and DB scenarios. As noted above, despite having overlap in inhibitory-dominant regimes (plots in S3 Fig) there were differences in input parameter balances that differentiated IVL states versus overly inhibited NIVL states. From close observation, we found that the pool mostly straddling a zero value EI metric was the one with low inputs (LLLL). This suggests that IVL states with low amounts of inputs will be more likely to have balanced excitatory and inhibitory inputs. We plot this pool on its own in Fig 3 for the two EI metrics and the AType+ and AType- models to show the likelihood of balanced states for low amounts of inputs.

### Moving closer to predicting synaptic inputs to IS3 cells *in vivo*

To determine what sort of inputs IS3 cells might receive *in vivo*, we performed a deeper exploration of our parameter sets. That is, despite already doing millions of simulations (see Methods), we performed additional simulations to probe and understand the robustness of our identified IVL states. We did this by obtaining a representative scenario from each pool that is robust, as determined by using a procedure that cycles through several random seeds. This procedure is described in more detail in the Methods. The synaptic input parameters for each of the obtained IVL representative scenarios are given in S6 Fig. We already know that 3 of the 16 pools did not produce IVL states (red squares), and in searching for robust, representative scenarios, another pool (LHLL) was removed (yellow square) for each of the AType+ and AType- models as a representative scenario was not found. We note that this pool actually had the smallest number of IVL states to begin with (see Fig 2A). Thus we have now reduced the possible IVL states to being from 12 pools and a representative scenario from each of these pools has been chosen. Additional considerations regarding the number of common inputs are described in the Methods (see S7 Fig).

**Fig 4:**
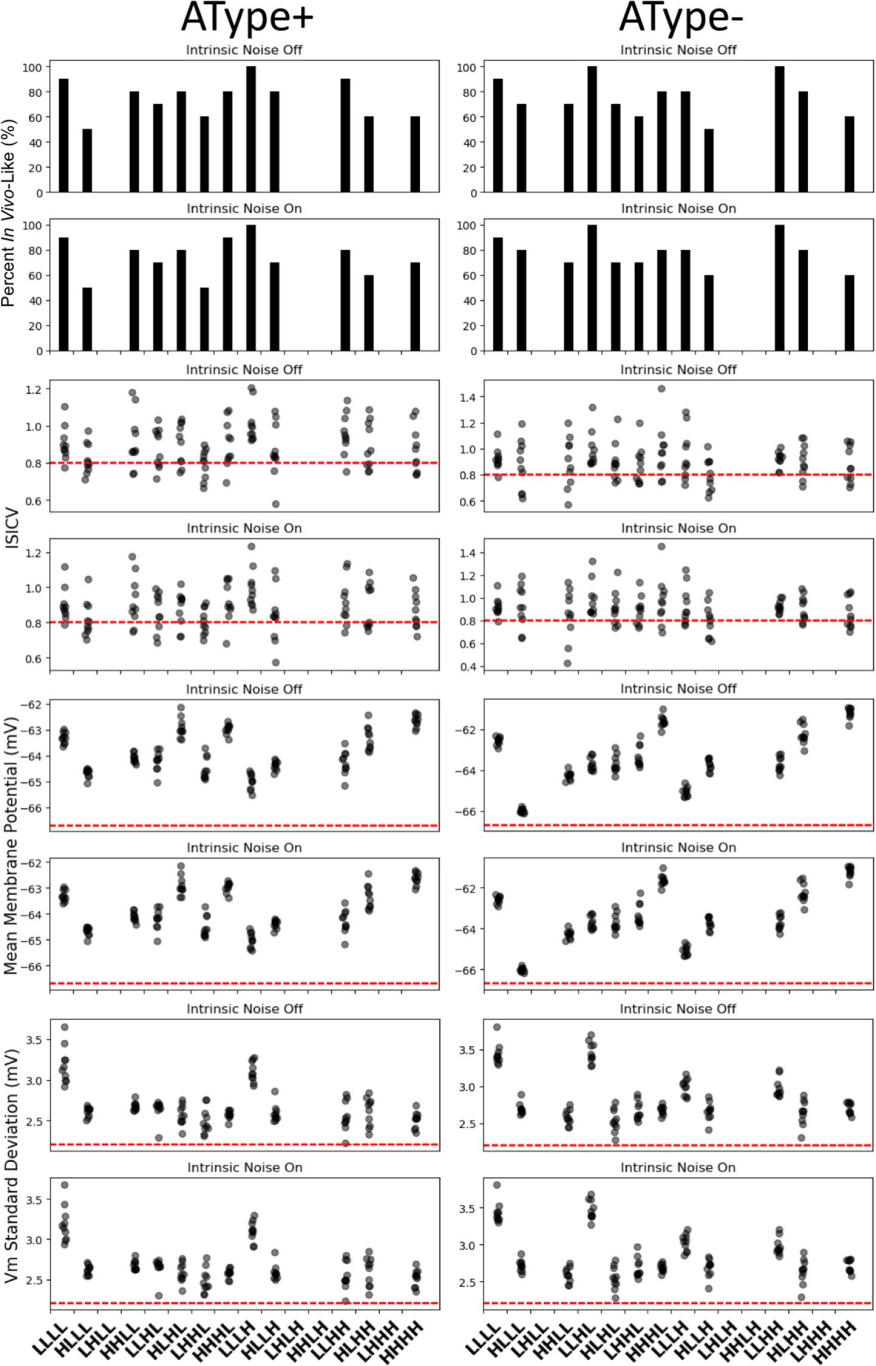
Consistency of IVL states for the representative scenarios with and without intrinsic noise. Panels of histogram plots, with or without intrinsic noise as labelled, for AType+ (left set) and AType- (right set) show % of simulations that each IVL representative scenario remained as a IVL state when re-run 10 times with 10 different seeds. Other panels: *ISICV*, mean *V*_*m*_, and standard deviation of the *V*_*m*_ for the 10 simulations of each representative scenario. Red lines indicate the threshold values used in the IVL metric. Consult Fig 2A for pool labels.

For each representative scenario we re-did the simulation ten times with a new set of different random seeds to examine the consistency of their IVL states (Fig 4). We dissected the consistency of the IVL states of the representative scenarios by plotting the different parts of the IVL metric along with the % of simulations that the IVL metric remained at three. We found that the representative scenario from pool LLLL was consistently IVL for both the AType+ and AType- models for most of the re-done simulations, and when it was not, it was due to the *ISICV* dipping a bit below the chosen threshold. This was also true for the representative IVL scenario from pool LLLH for the AType+ model and from pool LLHL for the AType- model. Also shown in Fig 4 is the same dissection, but without intrinsic noise, to ensure that this was not a determining factor. As described in the Methods, intrinsic noise is part of the IS3 cell model characteristics.

**Fig 5:**
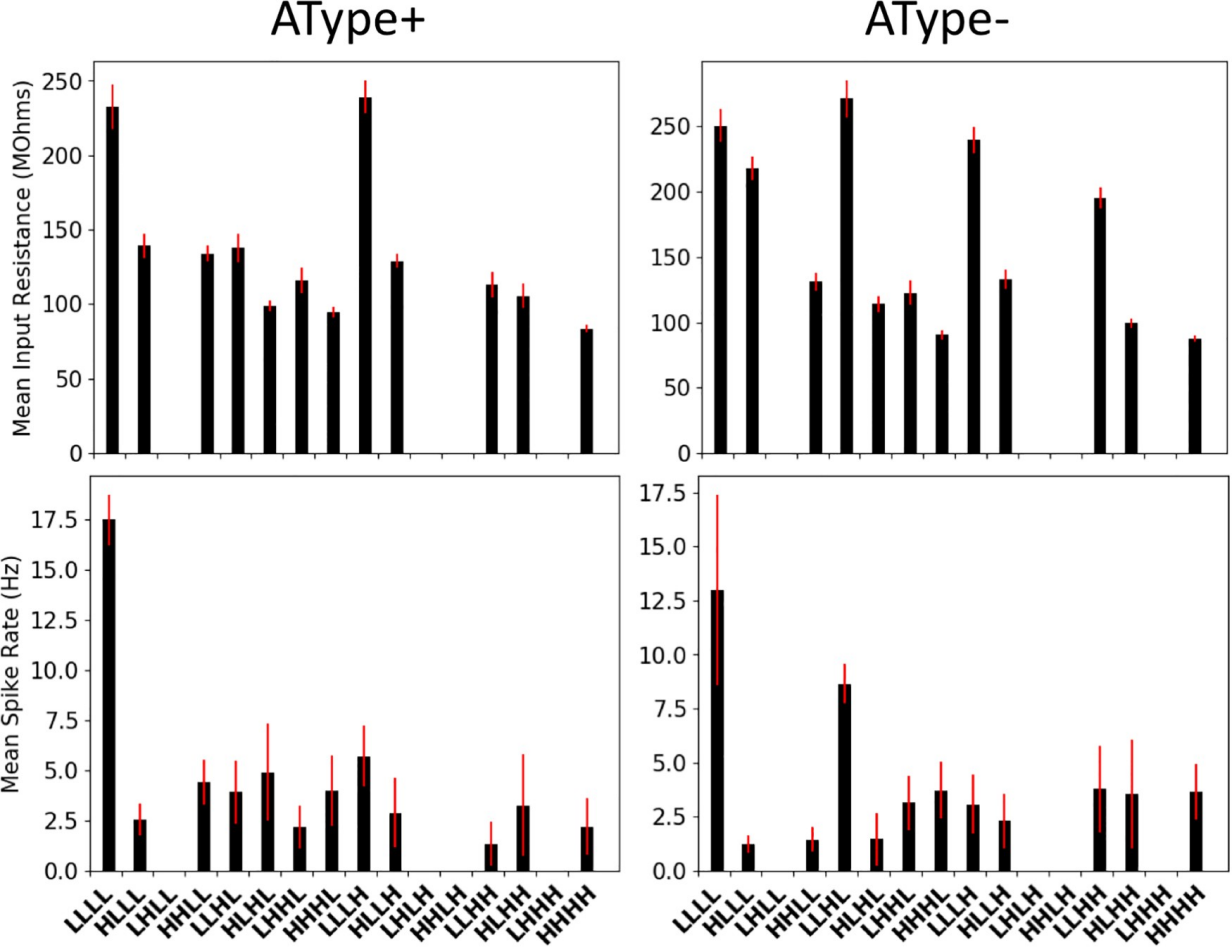
Input resistances and spike rates of IS3 cells in the representative IVL scenarios. **Top**: Plots of mean input resistance values, as computed across 10 simulations with different random seed values on each iteration. Standard deviations of the input resistance values are shown as red error bars. **Bottom**: Plots of mean spike rates, as computed across 10 simulations with different random seed values on each iteration. Standard deviations of the spike rates are shown as red error bars. When not receiving any synaptic input, models show no spiking and input resistance is: 388.71 MOhms (AType+), 406.57 MOhms (AType-).

In Fig 5 we show the input resistance and mean spike rates of IS3 cell models for the representative scenarios as re-done with the different random seeds. As expected, the input resistances were smaller compared to our model IS3 cells when not receiving synaptic input. Those specific values are given in the caption of Fig 5. Previous reports have indicated that there are decreases in input resistance during *in vivo* states [36, 40], which makes sense due to the larger amount of synaptic inputs being received *in vivo* relative to *in vitro*. Looking at the mean spike rates of the IS3 cells (bottom plots of Fig 5), we observed that they were larger when there were low amounts of inputs. This is also logical and consistent with our results above that showed that there was an inhibitory dominance with high amounts of inputs.

Interestingly, the four pools that had the most consistent IVL representative scenarios (LLLL for AType+ and AType-, LLLH for AType+, and LLHL for AType-) as identified in Fig 4 were those that had the largest input resistances and mean spike rates (see Fig 5). For the representative scenarios in other pools, changing the random seed had a larger impact on the consistency of the IVL state because the *ISICV* fell quite a bit below the chosen threshold of 0.8 on some of the trials, although we note that it has been demonstrated that *ISICV* values lower than our chosen threshold value of 0.8 can occur [41].

#### IVL states with low amounts of synaptic input have approximately equal balanced synaptic conductances

**Fig 6:**
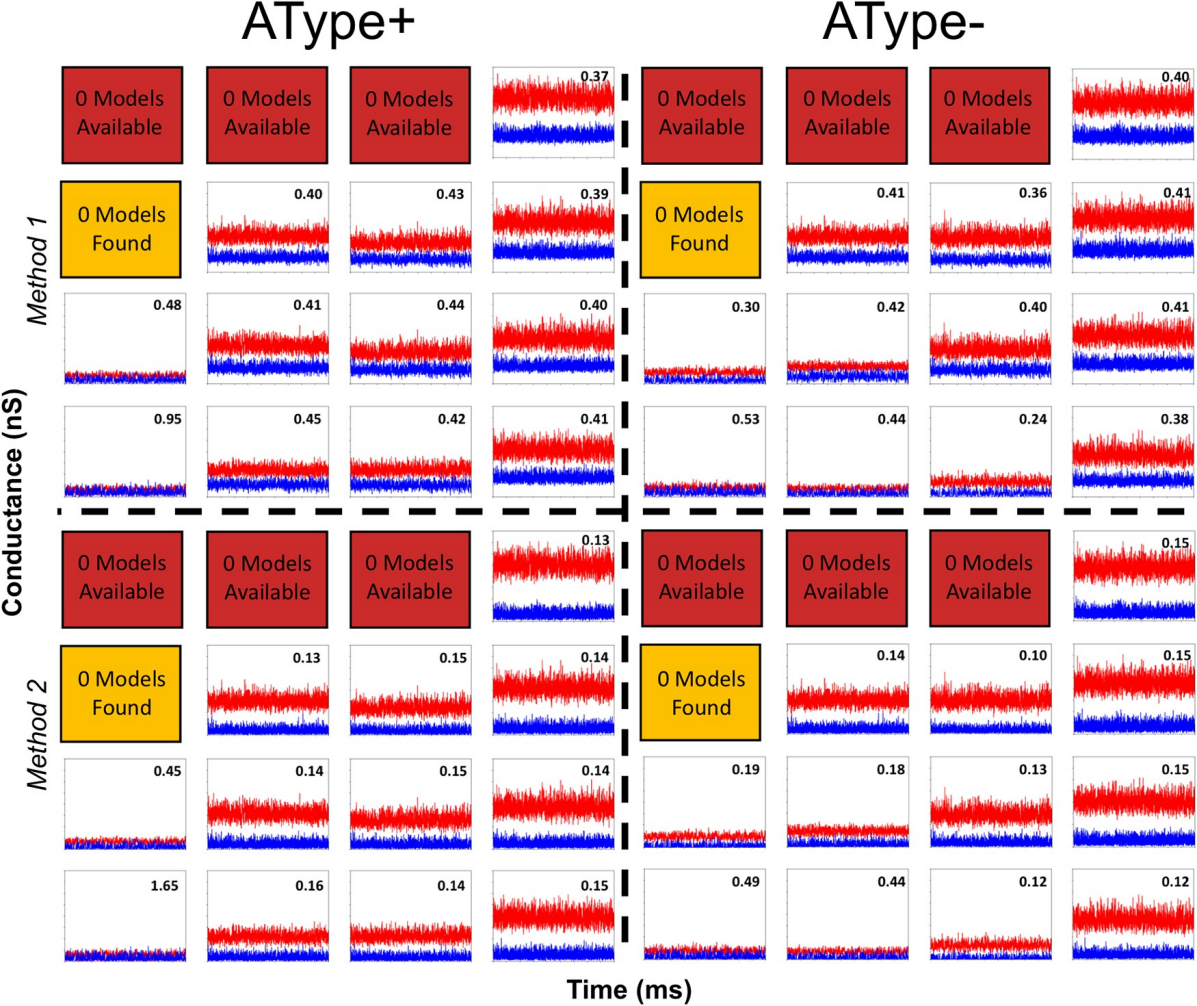
Conductance balances for IVL states using two different methods. Excitatory (blue) and inhibitory (red) conductances are shown for AType+ (left) and AType- (right) and as determined using either Method 1 (top) or Method 2 (bottom) for the representative IVL scenarios in each pool. For all plots: x-axis range is 1,000-10,000 msec; y-axis range is 0-16 nS. The numbers shown in each pool are the temporally averaged element-wise ratios of excitatory to inhibitory conductances. Method 1: Inhibitory or excitatory conductances recorded when excitatory or inhibitory inputs are respectively removed completely. Method 2: Inhibitory or excitatory conductances recorded when excitatory or inhibitory currents are not removed completely (i.e. the only measure taken is by changing the holding potential to their respective reversal potentials). Excitatory and inhibitory currents from which these conductances were computed can be seen in additional plots 10-11 on osf.io/6zg7a).

Though our EI metrics have shown that IVL states with low amounts of synaptic input are more likely to have balanced excitation and inhibition, our EI metrics do not include differences in distance-dependent synaptic parameters. Our inhibitory and excitatory synapses have different distance-dependent kinetics, as determined from optimized fits to experiment (see Eqs 3 and 4 in the Methods). Additionally, inhibitory synapses in our models have slower time constants than excitatory synapses. We thus directly examined the balance of excitation and inhibition by isolating excitatory and inhibitory currents (see Methods for details) in our IVL representative scenarios from the different pools and then converting them into conductances. In viewing these results, we found that the IVL representative scenarios for the pools with lower amounts of synaptic input (Fig 6, Method 1) exhibited more balanced magnitudes of excitatory and inhibitory conductances than those with larger amounts of inputs, which were all inhibitory-dominant. We quantified this by computing the mean of the ratio of excitatory and inhibitory conductances (E/I ratio), and this number is shown in each panel for the different pools.

It is interesting to consider potential confounding effects that would be present in experimental situations. That is, to separate out excitatory or inhibitory currents, one holds the cell at either the inhibitory or excitatory reversal potential respectively. Besides not necessarily knowing the appropriate reversal potential in experiment, one would typically have space clamp issues. Of course, we do not have these limitations in models so we can be sure about the excitatory and inhibitory conductances plotted in Fig 6 (Method 1). In Fig 6 (Method 2), we show the synaptic conductances that were obtained in our models if we separated excitatory and inhibitory currents as would be done experimentally. There was a clear difference between Method 1 and Method 2, and this is especially noticeable if one compares the quantified ratio numbers shown in each panel. Because the models were only clamped at the soma, inhibitory currents can still have an effect of reducing the current generated by excitatory synapses (i.e. space-clamp issues). This is most apparent when comparing the excitatory conductances which remain ‘glued’ to zero using the experimental approach which is a clear artifact (see Fig 6, Method 2). Since we did not have this artifact (with Method 1) we can clearly state that IVL states in our IS3 cell models when there is a low amount of synaptic input have approximately equally balanced excitatory and inhibitory conductances. Visually, this is easily seen to the case for the pools that were identified as having the most consistent IVL representative scenarios above in Fig 4. Namely, LLLL for AType+ (E/I ratio = 0.95), LLLL for AType- (E/I ratio = 0.53), LLLH for AType (E/I ratio = 0.48) and LLHL for AType- (E/I ratio = 0.44). Having a larger amount of synaptic input required a shift towards an inhibitory dominance to maintain an IVL state. Again, this is easily visualized in Fig 6, and also quantified in the smaller numbers for the excitatory to inhibitory ratios.

### IS3 cells need to have balanced excitation and inhibition to exhibit sensitivity to theta-timed inputs

**Fig 7:**
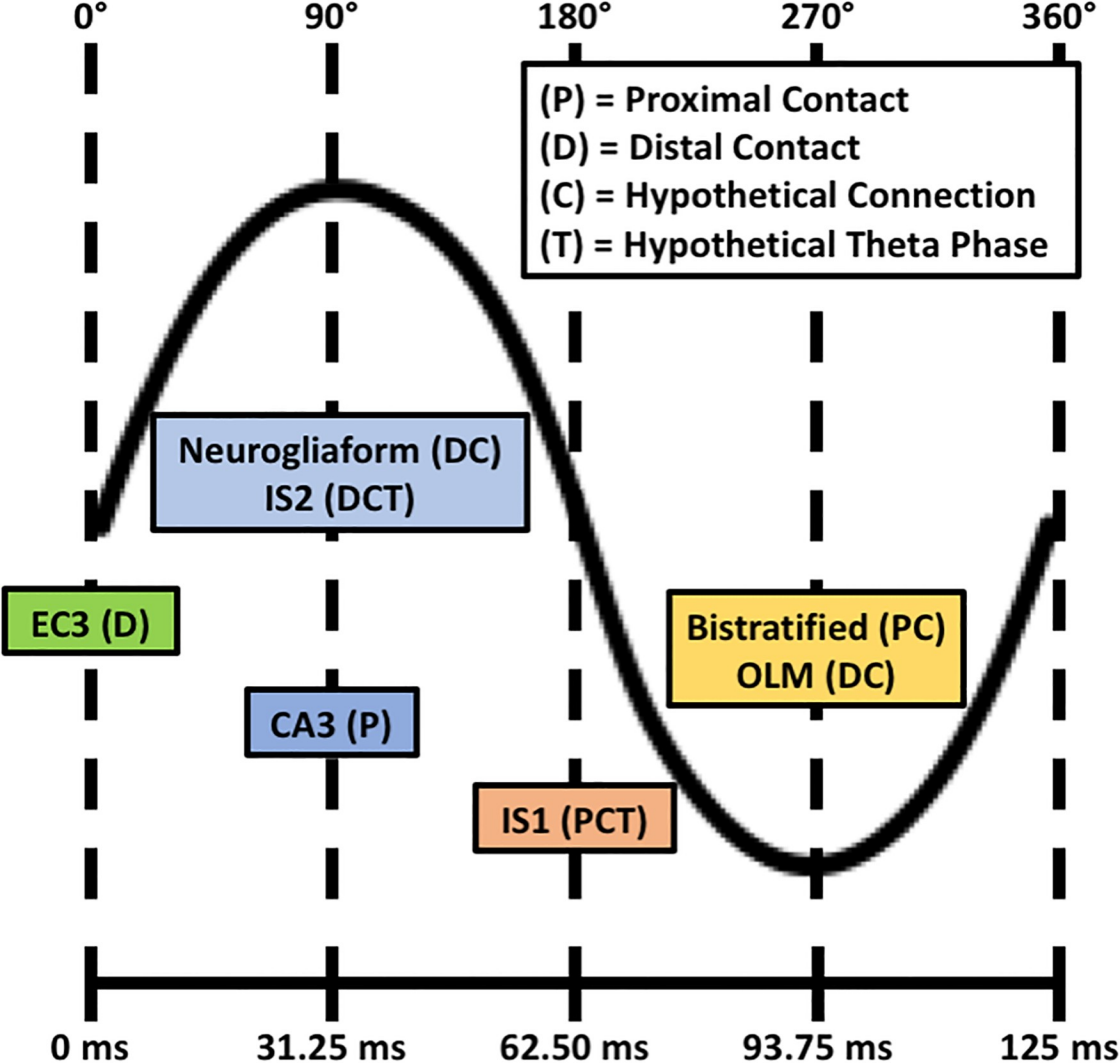
Schematic of timings of excitatory and inhibitory input populations. The relative timing of excitatory input populations (i.e. EC3 and CA3) and hypothetical local inhibitory input populations (e.g. bistratified, OLM, IS1, IS2, and neurogliaform) to IS3 cells is shown. The legend denotes inputs that would connect to IS3 cell proximal dendrites (P), inputs that would connect to IS3 cell distal dendrites (D), connections that are hypothetical (i.e. non-confirmed; C), as well as inputs with relative timing during the theta-cycle phase that are hypothetical (T). For the input populations with hypothetical relative theta-cycle timing, we estimated that they would spike 1/4 of a phase after either CA3 or EC3 input populations spike, depending on where the dendrites of those cell types are likely to be positioned. EC3 = entorhinal cortex, layer III pyramidal cells; OLM = oriens lacunosum moleculare interneurons; IS1 = interneuron specific, type 1 interneurons; IS2 = interneuron specific, type 2 interneurons.

The lack of *in vivo* recordings of IS3 cells means that it is unknown how they might contribute to brain function. However, given their connections to other inhibitory cell types that are involved in theta rhythms, and that they can preferentially influence OLM cells at theta frequencies *in vitro* [19], it is reasonable to hypothesize that IS3 cells need to be responsive to theta-timed inputs. To examine this, we implemented theta-timed layer-specific inputs at 8 Hz. In order to define these theta-timed input populations, we approximated synapse numbers (see Methods) as well as the relative timing of both known and hypothetical excitatory and inhibitory cell type inputs to IS3 cells during a theta-cycle [42, 43] (Fig 7).

**Fig 8:**
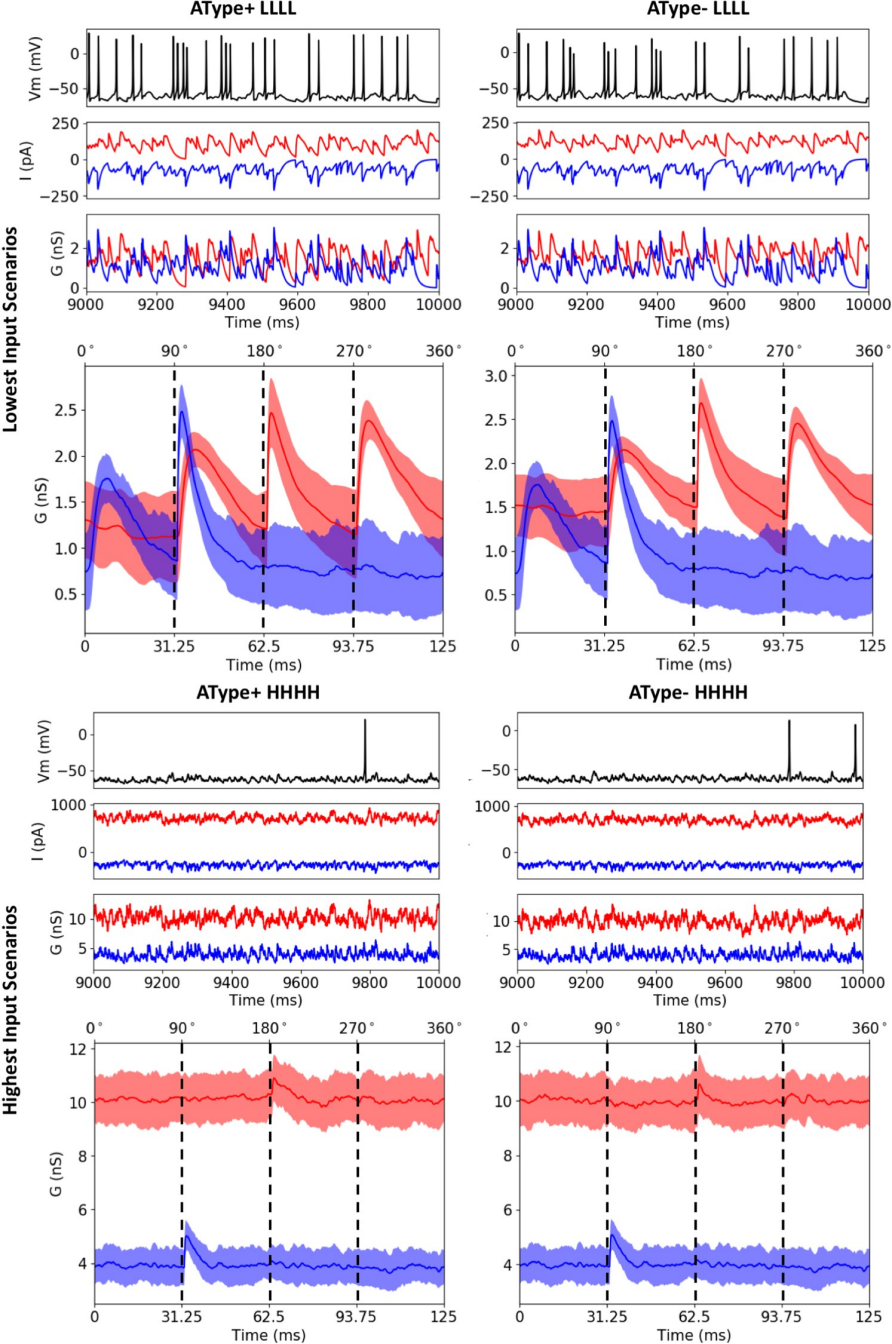
Illustrations from two scenarios with theta-timed inputs. Lowest (LLLL) and highest (HHHH) amounts of inputs are shown using the IVL representative scenario. AType+ models are shown on the left and AType- on the right. For each scenario, the top plots show zoom-ins of voltage traces, and inhibitory (red) and excitatory (blue) currents and conductances during the addition of theta timed inputs. Conductances and currents for all IVL representative scenarios are shown in an additional plot 11 on osf.io/6zg7a. The bottom plots show example average inhibitory (red) and excitatory (blue) conductance traces during one theta cycle. We took the conductance traces (1,000 to 10,000 ms), split them each into their 72 theta cycles (i.e. 9000 *ms*/125 *ms* = 72 *cycles*), and then computed the average 125 ms theta cycle traces for each representative scenario. Shaded areas show the amount of standard deviation above or below the mean. In the LLLL scenario, clear peaks in conductance can be observed for when theta-timed inputs are occurring at 0°, 90°, 180°, and 270°.

**Fig 9:**
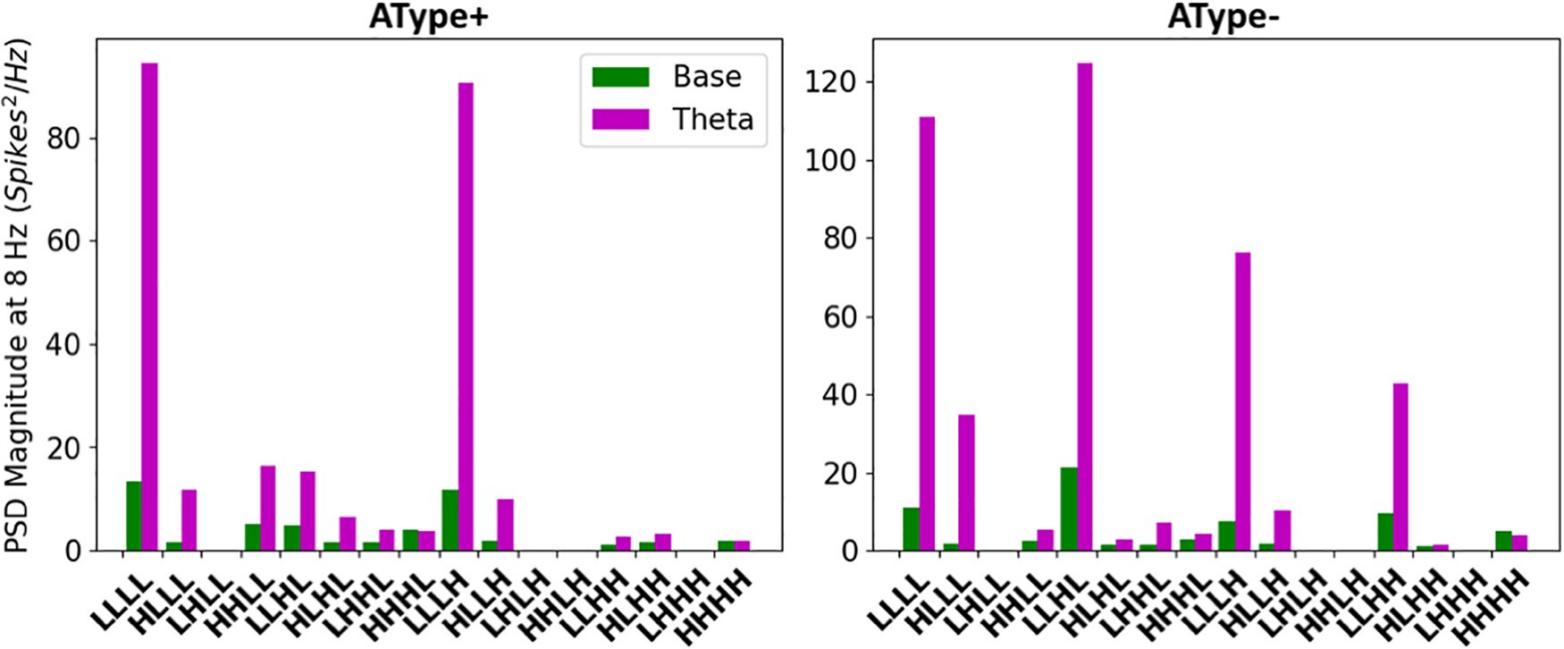
Power spectral densities (PSDs) for different inputs with theta-timed inputs. PSDs computed from the spike trains of IVL representative scenarios before (green) and after (magenta) the addition of theta-timed (8 Hz) inputs for the different input pools.

In Fig 8 we show IS3 cell output when theta inputs were added for low (LLLL) or high (HHHH) amounts of synaptic input IVL representative scenarios. Also shown are the excitatory and inhibitory currents and conductances, computed using Method 1 (see Fig 6). We found that that the scenarios with smaller amounts of inputs exhibited more rhythmicity as compared to scenarios with larger amounts of inputs. This was borne out when computing the power spectral densities (PSDs) of the spike trains before and after the addition of theta-timed inputs, as shown in Fig 9. The representative scenario from pool LLLL (i.e. lowest amount of inputs) showed a much more appreciable increase in the PSD at 8 Hz when compared to the representative scenario from pool HHHH. As well, when looking at averaged excitatory and inhibitory conductances during a theta cycle, the low input representative scenario was more responsive to theta-timed inputs (Fig 8). As might be expected, excitation led inhibition at the quarter cycle due to its more proximal input locations (Fig 7) and faster synaptic time constants. Inhibitory or excitatory peaks at other quarter cycles were also clearly apparent in the representative scenario from pool LLLL. To ensure the robustness of our results, we re-did these theta-timed simulations for the representative scenario from pool LLLL five times and the averaged voltage output from them are shown in S9 Fig.

These results suggest that the synaptic inputs in the baseline IVL scenarios with larger amounts of inputs are drowning out the influence of the comparatively smaller theta-timed inputs. This can be appreciated by noting that the IVL representative scenario with highest input resistance also responded the most to theta-timed inputs (Fig 5, top plots; see LLLL and LLLH for AType+; see LLLL, LLLH, and LLHL for AType-). Additionally, having inhibitory dominance when there are larger amounts of inputs will decrease neuron sensitivity to new input by increasing the threshold for spiking. In line with this, we observed considerably larger spike rates in the representative IVL scenario with low amounts of inputs (Fig 5, bottom plots; see LLLL for both models). In summary, our findings demonstrate that having low amounts of inputs specifically in terms of number of synapses, in which there are approximately equally balanced inhibitory and excitatory conductances, allow our IS3 cell models to exhibit IVL states as well as to be sensitive to additional theta-timed inputs.

## Discussion

### Summary and predictions

In this work we explored synaptic input parameter spaces that generate *in vivo*-like states for IS3 cell models. We found that when there is a sparse amount of common synaptic inputs with approximately equally balanced excitatory and inhibitory conductances, *in vivo*-like states are present as well as a sensitivity to additional theta-timed inputs. Further, there were differences depending on whether the IS3 cell model had A-type potassium channels in their dendrites (see next section). It was possible to have *in vivo*-like states with a larger amounts of inputs, but then there was a stronger inhibitory dominance and the sensitivity to additional inputs was lost. We found this to be due to lower input resistances with the larger synaptic input currents. It is interesting to note that IS3 cells have been found to have high input resistances relative to other cell types [19]. If IS3 cells are to preferentially control other inhibitory cell types at theta frequencies *in vivo* as they do *in vitro* [19], then it is reasonable to expect that IS3 cells need to be able to be recruited with preferred firing at theta to be able to exert this influence. Therefore, we predict that IS3 cells *in vivo* have inputs with approximately equally balanced conductances. We note that the vast majority of studies regarding *in vivo* excitatory-inhibitory balances have been in cortical circuits. In the hippocampus [44], there is a balancing of excitation and inhibition on pyramidal cells during gamma oscillation amplitude modulations. In cultured cells, a local structural excitation/inhibition balance on hippocampal pyramidal dendrites has been shown [45]. Overall, our work suggests that baseline, balanced states exist in hippocampal circuits *in vivo*.

Our work suggests several experimentally testable predictions. We have predicted that baseline synaptic inputs *in vivo* need to be sparse and balanced in order to be sensitive to additional theta-timed inputs. This can be verified using dynamic-clamp techniques *in vitro*, though this would ignore the effects of dendritic signal propagation. Ideally, in order to establish whether such conditions are realistic, one would need to perform experiments similar to what was done for cortical pyramidal cells [36], but with IS3 cells. However, such experiments would be extremely difficult for IS3 cells *in vivo* given the location of the hippocampus deep inside the skull. We have also assumed that IS3 cells will respond to theta-timed inputs, as they receive inputs from excitatory neuronal populations that have been shown to spike rhythmically during theta rhythms [43]. We also note that since IS3 cells receive inputs from CA3, they are likely to be activated during sharp-wave associated ripples, and this can be examined *in vivo*. As mentioned, direct patch clamp recording of IS3 cells cells *in vivo* would be highly challenging to test this. An alternative would be to perform juxtacellular recordings or calcium imaging experiments. Efforts on the latter are already underway [20].

### Intrinsic properties and comparison to pyramidal cells

That the synaptic inputs could be low and still have an IVL scenario is suggestive of the importance of *both* intrinsic and synaptic homeostatic balances of IS3 cells in their contribution to hippocampal circuitry. In our explorations we used two IS3 cell model variants from our previous work that captured IS3 cell features [37]. They were shown to have differences in their responses to increasing levels of synaptic bombardment. Due to its intrinsic properties, the AType- model (A-type potassium channels restricted to soma) generated larger numbers of *in vivo*-like states than the AType+ model (A-type potassium channels present in dendrites), as well as a larger number of models nearing depolarization block. Although at present we do not know the distribution of A-type potassium channels in IS3 cells, their presence or absence in IS3 cell dendrites clearly would affect cell excitability. Further, our IS3 cell models currently have a minimal set of ion channel types and other channel types could clearly make additional contributions. For example, calcium channels are not included in our models even though there is evidence for them [46], and they could potentially be distributed to more distal IS3 cell dendrites. If included, dendritic calcium channels could potentially serve to help amplify synaptic inputs and possibly shift the high input inhibitory-dominant IVL regimes towards more balanced regimes. In essence, our prediction of low synaptic inputs *in vivo* highlights the importance of having a biophysical characterization of ion channels in IS3 cells.

Pyramidal cells have been studied much more extensively *in vivo* [36, 47, 48] relative to inhibitory cells or specifically IS3 cells. Relative to their hippocampal counterparts, IS3 cells have much smaller synaptic densities [49, 50], and thus we would expect pyramidal cells to have even lower input resistances *in vivo* due to larger synaptic densities. However, pyramidal cells are considerably larger than IS3 cells and so would have smaller axial resistances, which would promote signal propagation and better counteract the decreases in input resistance. As well, pyramidal cell dendrites have several active voltage-gated channels spread throughout the further lengths of their dendrites [51]. This would also promote signal propagation along pyramidal cell dendrites as compared to IS3 cells, which only have spike-propagating active properties in proximal dendrites as modelled here.

### Theoretical and biological balances

“Balance” in the context of excitation versus inhibition, has been used in several ways. In some instances balance has been used to define coincident increases in conductance, for example during rhythms, where transient increases in excitation can be coupled with transient increases in inhibition. Previously this has been termed a ‘tight balance’ [3], and does not necessarily imply that the levels of excitation and inhibition are equal. There is also a ‘loose balance’ terminology [3], that would be reflective of fluctuation-driven states [52].

Although principles of brain coding remain to be clearly deciphered, they involve firing rates, spike timing and correlations, and repeating patterns in various capacities [53]. The variability of neural firing increases information content and theoretical studies have shown that networks with loosely balanced excitation and inhibition exhibit irregular spiking or an asynchronous state [54]. Correlations in these balanced networks can vary depending on spatial connectivities [55]. Neurons would be considered to be in a fluctuation-driven regime, as opposed to a mean-driven regime [52], with spike firing occurring randomly due to a loose balance between excitation and inhibition. From a coding perspective, efficiency and information content favours having neither an excitatory nor an inhibitory dominance [2, 3]. Further, having a loose or global excitatory/inhibitory balance is thought to promote rate coding [2, 3, 56] and having a tight balance would facilitate spike time coding. Tight balances between excitation and inhibition have been observed *in vitro* [57], during rhythmic contexts [44, 58], as well as *in vivo* [40, 48, 59]. How tightly excitation and inhibition are balanced *in vivo* would depend on the specificities of the anatomical circuit and the behavioural context. From an individual cell perspective, experimental studies indicate that excitation and inhibition are proportionally balanced *in vivo* [60], although there could also be an inhibitory dominance [61]. However, extracting these balances from experiment can be tricky due to space clamp and reversal potential estimations. We directly showed the effect of space clamp in excitatory and inhibitory balances in our explorations here where excitatory conductances were underestimated.

In turtle lumbar spinal circuits, it has been reported that neurons spend a component of their time in fluctuation-driven regimes and another component in mean-driven regimes [62]. Neurons in fluctuation-driven regimes were found to have enhanced sensitivity, whereas neurons in mean-driven regimes had reduced sensitivity. This highlighted a network-level ‘Goldilocks Zone’ where neurons could be both stable and sensitive to new inputs [63]. Our *in vivo*-like states are suggestive of such a scenario in hippocampal circuits as we have found fluctuation-driven regimes with enhanced sensitivity to additional inputs for IS3 cells. Increased excitation would move one toward a mean-driven regime. We achieved our *in vivo*-like state using a loose balance between excitation and inhibition, but tight balances are warranted when introducing a rhythmic or transient context such as during theta rhythms or sharp wave-associated ripples [42, 58, 64].

The prominent theta rhythm in the hippocampus, associated with spatial navigation and memory consolidation, is observed during REM sleep and movement [65, 66]. In CA1 this rhythm is generated through a combination of external inputs from entorhinal cortex and CA3 [43, 67], recurrent inputs from medial septum [68], as well as intrinsically [69] through inhibitory cell interactions [58, 70]. During intrinsically generated theta rhythms in CA1, there is a tight balance between excitation and inhibition for different neuron types [58]. In our explorations we found that common inputs were important in generating *in vivo*-like states for our IS3 cells as there was a noticeable increase in the size of the parameter spaces that generated *in vivo*-like, fluctuation-driven regimes when common inputs were present. This indicates that correlations are generically present in hippocampal microcircuits, as is already known for cortical circuits [3]. Our prediction of evenly balanced excitatory and inhibitory conductances and of *low* amounts to IS3 cells confers high input resistances and sensitivity to additional theta-timed inputs.

### Balance and disease states

In considering disease states, a consideration of excitation/inhibition balance on its own is over-simplistic [71]. For example, it was recently shown in a mouse model of Fragile-X Syndrome that correlations and firing rate changes were insufficient to capture the shifts seen in the population activities with disease when comparing a spiking circuit model with analyses of calcium imaging data [72].

While we found balanced regimes representative of neural states in live animals, we also highlighted inhibitory-dominant and excitatory-dominant regimes in our models that could be present during disease states. For example, at many levels of inputs (i.e. the different parameter range pools), excitatory-dominant input scenarios can generate models nearing depolarization block, thus demonstrating that excitatory shifts in balance could lead to inactivation of particular cell types via depolarization block if given enough excitation. This was suggested to be the case for fast spiking interneurons during the spread of epileptic waves across cortex [73–75]. Interestingly, inhibitory drive from IS3 cells has been shown to be reduced in a pilocarpine-induced model of epilepsy [76], suggesting a shift towards excitatory-dominant, depolarization block-generating regimes in IS3 cell postsynaptic targets.

### Limitations

Although we have done an extensive exploration to examine how much and what balance of excitatory and inhibitory synaptic inputs IS3 cells might receive *in vivo*, we did limit our exploration by grossly separating proximal and distal excitatory inputs and not controlling the spatial locations of common input. However, that we separate proximal and distal locations at all will allow us to examine how inputs from different regions could control IS3 cells during different behavioural states. We based our IVL metric from other cell types since *in vivo* information is not available for IS3 cells directly although our consideration of threshold values did take IS3 cell specifics into consideration. While changing the specific threshold values used in our IVL metric would change the details of our results, we do not expect that our general results that predict low amounts of equally balanced excitation and inhibition to change considering our examination of unpacking the components of the IVL metric.

We used a simple first-order synaptic kinetic model to simulate our postsynaptic effects. Although there is evidence that short-term synaptic depression exists for inputs onto VIP+ cells in neocortex [32], it is unknown whether this is also the case for VIP+ IS3 cells in hippocampus. Given the high levels of random activity in our simulations, it is unclear how implementing synaptic depression would change our results, but it is likely that the overall synaptic currents and conductances generated during higher spike rate input regimes will be smaller. As well, in our synaptic model we assume that there is a 0% failure rate (i.e. all common synapses succeed at transmitting an EPSC/IPSC at the same time), which is unlikely to be the case. If implemented, this would probably reduce the number of IVL scenarios being generated, as failure rates would decrease the probability of postsynaptic spiking. Finally, we have noted that our cellular models are limited in the types of ion channels incorporated, but their development ensured that IS3 cell features were captured [37].

### Concluding remarks and future work

While excitation and inhibition balance is central to brain coding, it is important to reconcile the specifics associated with theoretical insights and biological realities. By doing an extensive computational exploration here we have been able to *both* predict synaptic inputs to IS3 cells *in vivo* as well as to unpack some of these ‘balance’ aspects.

Using our predicted *in vivo*-like states we will investigate the conditions and contexts under which IS3 cells can be recruited to spike and contribute to theta rhythms and sharp wave-associated ripples in the hippocampus. Ongoing experimental work shows promise in recording from IS3 cells *in vivo* [20] and our modeling work can help guide such studies. In combination, we aim to achieve an understanding of how IS3 cells could critically contribute to hippocampal activities and behaviours. As IS3 cells specifically target OLM cells, we also aim to explore *in vivo*-like states in previously developed OLM cell models [77] and create *in vivo*-like microcircuits. Similar to cortex, perhaps IS3 cells “open holes in the blanket of inhibition” [31] to bring about and control theta rhythms and sharp wave-associated ripples in the hippocampus.

## Methods

### IS3 cell models

We used multi-compartment model variants of IS3 cells developed in our previous work [37] using the NEURON software environment [78]. The morphological and electrophysiological properties of the models are directly based on IS3 cell recordings. The model consists of 221 somatic, dendritic, and axon initial segment compartments (S1B Fig), and exhibits firing frequency and depolarization block characteristics of IS3 cells. The ion channel types and distributions in our IS3 cell models reflect interneuron specific characteristics as channel data specific to IS3 cells is not available at present. They include transient and persistent sodium channels and A-type and delayed rectifier potassium channels. Also, to reflect subthreshold activities observed in IS3 cells, intrinsic noise was included as done in our previous models [79]. Although it is expected that there are more ion channel types in IS3 cells, we did not include any additional ones in building these models, but rather focused on a minimal set since this was sufficient to capture IS3 cell features. Direct immunological evidence was obtained for delayed rectifier potassium channels in dendritic portions of IS3 cells but it is at present unknown about other channel types [37]. Here, we used two of our models that captured IS3 cell electrophysiological features, one that has A-type potassium channels in the dendrites (AType+) and one that does not (AType-). The specifics of these models with their somato-dendritic ion channel distributions are given in Table 3.

**Table 3:** Intrinsic properties of IS3 cell model variants. *G_Na,t_* = transient sodium channel conductance; *G_Na,p_* = persistent sodium channel conductance; *G_Ka_* = A-type potassium channel conductance; *G_Kdrf_* = fast delayed rectifier potassium channel conductance.

Model	Distribution	*G_Na,t_* (mS/cm^2^)	*G_Na,p_* (mS/cm^2^)	*G_Ka_* (mS/cm^2^)	*G_Kdrf_* (mS/cm^2^)
AType+	Uniform across soma and first 70 *μ*m of dendrites (*G_Na,p_*: soma only)	70	0.075	70	250
AType-	Uniform across soma and first 70 *μ*m of dendrites (*G_Na,p_* and *G_Ka_*: soma only)	55	0.15	30	295

### Synaptic model and parameters

We used excitatory and inhibitory synaptic parameters that were obtained from optimal fits to the experimental data [80]. We used NEURON’s Exp2Syn synapse model, which describes synapses as two-state kinetic schemes:

$$\begin{array}{c} i=G(V-E_{R}) \end{array}$$ (1)

$$\begin{array}{c} G=weight\times factor\times (exp(-\frac{t}{\tau_{d}})-exp(-\frac{t}{\tau_{r}})) \end{array}$$ (2)

Where *i* is the synaptic current, *G* is the synaptic conductance, *E* is the reversal potential, *V* is the membrane potential, *weight* is the synaptic weight, *factor* is a NEURON process that is used to normalize the peak synaptic conductance to the value of *weight*, *t* is time, *τ*_*r*_ is the rise time, and *τ*_*d*_ is the decay time. Both model variants (AType+, AType-) were used in obtaining synaptic parameters as derived from optimizations that fit passive model responses to those seen in layer-specific-evoked excitatory postsynaptic currents (EPSCs) and spontaneous inhibitory postsynaptic currents (IPSCs) [80]. The layer-specific evoked EPSCs were obtained under minimal stimulation. Evoked EPSCs generated from stimulation in stratum radiatum were assumed to be Schaeffer collateral inputs from CA3 to IS3 cell proximal dendrites. Evoked EPSC responses generated from stimulation in stratum lacunsom-moleculare were assumed to be inputs from entorhinal cortex layer III (ECIII) to IS3 cell distal dendrites [81].

For excitatory synapse models, we applied a reversal potential of 0 mV, and applied a linear distance-dependent weight rule, according to the weight of the best fit for proximal dendrites (i.e. <300 *μ*m from the soma), and the weight of the best fit for distal dendrites (i.e. >300 *μ*m from the soma). This border was based on the layout of the dendritic morphology of our reconstructed models, with proximal dendrites in the Stratum Radiatum (SR), and distal dendrites extending to the Stratum Lacunosum Moleculare (SLM). Excitatory rise and decay time constants (i.e. *τ*_*r*_ and *τ*_*d*_) for either proximal or distal synapses were then fixed to the optimized time constants of the best fit in proximal or distal dendrites, respectively.

weight=0.00230814nS/μm×distance+0.22016666nS (3)

$$\begin{array}{c} \tau_{r_{proximal}}=2.9936\times 10^{-4},\;\;\tau_{d_{proximal}}=2.4216,\\ \;\;\tau_{r_{distal}}=6.1871\times 10^{-4},\;\;\tau_{d_{distal}}=3.1975\;ms \end{array}$$

For the inhibitory synapse models, we applied a reversal potential of -70 mV, and applied a linear-distance-dependent weight rule, according to the optimized weight values of the two best fits, regardless of their synaptic locations. Inhibitory time constants for all synaptic locations in the model were then fixed to the optimized time constants of the best inhibitory synapse fit.

$$\begin{array}{c} weight=0.00469125\;nS/\mu m\times distance+0.2695779\;nS \end{array}$$ (4)

$$\begin{array}{c} \tau_{r}=0.1013,\;\;\tau_{d}=4.8216\;ms \end{array}$$

More details on synaptic parameter optimizations are given in [80].

### Parameter exploration ranges for excitatory and inhibitory synapses, strategy and setup details

In our parameter explorations, we kept the individual synaptic amplitude and time course parameters (i.e. weight, rise times, and decay times) fixed, see above section. However, we varied the inhibitory and excitatory spike rates and numbers of synapses (i.e. four parameters in total). Since these parameters are variable *in vivo*, depending on the context and the neuron type of interest, we wanted to explore full ranges of possible input parameter combinations, based on what is known experimentally.

*Excitatory spike rates*: We first looked at the spike rate distributions of CA3 pyramidal cells, as well as ECIII pyramidal cells. While CA3 pyramidal cells tend to occasionally show high spike rate distributions (i.e. >100 Hz) during anaesthesia, which could correspond to bursting activity [82, 83], on average, spike rates tend to be below 30 Hz, with peak firing rates occurring during behavior while the animal moves through place fields [84] or time fields [85]. Similarly, pyramidal cells in ECIII (i.e. in both lateral and medial ECIII) exhibit below 30 Hz spiking during 200 pA stimulation *in vitro* [86, 87], as well as during grid field traversals *in vivo* [88]. Considering these observations, we set maximum excitatory spike rates of 30 Hz for both proximal and distal excitatory inputs.

*Inhibitory spike rates*: Though the spike rates of fast-spiking parvalbumin-positive (PV+) cells *in vivo* during theta or low oscillations tend to be below 40 Hz, during sharp wave-associated ripples these tend to spike at up to 160 Hz [23]. Since it is unclear which groupings of local CA1 inhibitory neurons target IS3 cells and we were simulating baseline *in vivo* states (i.e. without sharp-wave associated ripples), we set the maximum inhibitory spike rate to be 100 Hz.

*Synapse numbers*: The maximum synapse numbers in the model were deduced from excitatory and inhibitory synaptic densities onto calretinin-positive (CR+) cells in hippocampal CA1 [49]. Using these maximal densities, as well as the average length of each compartment in the IS3 cell model, we estimated there to be approximately 9 excitatory synapses and 2 inhibitory synapses per compartment. In total this estimated maximums of 1530 excitatory synapses and 344 inhibitory synapses spread across the dendritic arbor of the model.

We also investigated whether synaptic inputs were common or independent (S1A Fig). Inputs that are common means that a presynaptic input forms multiple synaptic inputs onto the IS3 cell model, and thus these common inputs are assigned identical spike trains. Independent inputs, simply means that each synapse has a unique spike train (i.e., a different random seed is used). For common input parameter searches, we used numbers of 9 common excitatory inputs and 4 common inhibitory inputs, although different numbers were also examined (see below). These numbers were chosen because a) they fell well within ranges of common inputs seen in somatosensory cortex according to findings by Blue Brain Project [89, 90], and b) they allowed a high parameter search resolution based on the maximal excitatory and inhibitory synapses. That is, if looking at larger numbers of common inputs, the parameter search resolution would become smaller because each time we would increase the number of synapses we would have to add an amount of synapses equal to or divisible by the number of common inputs. Note that the maximum numbers of excitatory and inhibitory synapses are 1530 and 344 respectively, which are exactly divisible by 9 and 4. The parameter ranges explored in our parameter searches are shown in Table 4.

**Table 4:** Parameter exploration ranges. Output from a total of 4,502,960 ten-second simulations are obtained when considering all possible parameter combinations and the two model variants.

Parameter	Minimum	Resolution	Maximum
Excitatory Spike Rate (Hz)	0	5	30
Inhibitory Spike Rate (Hz)	0	10	100
Number of Excitatory Synapses	18	18	1530
Number of Inhibitory Synapses	4	4	344
Excitatory Common Inputs	1	-	9
Inhibitory Common Inputs	1	-	4

We performed our parameter search in the following manner. The locations of inhibitory and excitatory synapses were chosen randomly but with proximal and distal locations having an equal number of excitatory synapses. Throughout our parameter explorations, we adjusted the number of synapses by selecting synapses and assigning them spike trains. Each presynaptic spike train was obtained by sampling spike times randomly from a uniform distribution. The mean frequency (spike rate) of the presynaptic spike train was set by ensuring that there was an appropriate number of spikes for the length of time of the simulation. For example, for 100Hz, 1,000 spike times would be sampled for a 10 second simulation. This presynaptic spike rate was adjusted by changing the number of spike times being sampled. Further implementation details are provided in the next section. S1B and S1C Fig show an example for one set of parameters—8 inhibitory synapses activated with 30 Hz presynaptic spike trains, and 144 excitatory synapses activated with 5 Hz spike trains. S1B Fig shows the random distribution of 8 inhibitory (red), 72 proximal excitatory (blue) and 72 distal excitatory (green) synapses on the dendritic tree of the IS3 cell model, and S1C Fig shows a raster plot of the presynaptic spike trains. In this example set of parameters, the common input scenario (S1 Fig) is shown.

### Implementation details

The synapse locations were determined as follows: The 1530 excitatory and 344 inhibitory synapses were fully distributed throughout the dendritic tree with 9 excitatory and 4 inhibitory synapses per compartment. To activate the given number of synapses being explored, vectors of presynaptic spike times were assigned to the selected synapses using NEURON’s VecStim function. Non-assigned synapses remained silent over the course of the simulation since they did not receive any input spike trains. The locations of the active synapses were determined using a customized function for random index selection, which chose indices by sampling integers from a discrete uniform distribution. In this function each group of synapses was organized in vectors (proximal excitatory, distal excitatory, and inhibitory). For each synapse vector a random indexing vector of the same length was created where the content of each index (in the random indexing vector) is an integer value sampled using NEURON’s random.discunif(min,max). A loop that keeps sampling from random.discunif(min,max) was done until every number in the random indexing vector is unique, so that synapses were not represented more than once.

Each presynaptic spike train was determined by sampling spike times from a uniform distribution of length 0 to 10,000 msec (the simulation length that we used). We used NEURON’s default random number generator, which is a variant of the Linear Congruential Generator combined with a Fibonacci Additive Congruential Generator (ACG), to sample from a discrete uniform distribution. The spike rate was adjusted by changing the number of spike times being sampled within this time window. Once the spike times were selected and sorted, we used NEURON’s VecStim function to assign the custom-built spike time vectors into the different synaptic locations. The spike trains were irregular and our spike rates were given by # of spikes/10 (Hz), since our simulation lengths are 10 seconds. We emphasize that it is not the interspike intervals being sampled from a uniform distribution, but the spike times. Interspike intervals are commonly thought to follow a negative exponential distribution (though there are exceptions to this rule [91]). While we did not know what interspike interval distributions are followed by inputs to IS3 cells, we note that when data is randomly sampled from a uniform distribution and then sorted in ascending order, the intervals between the data tend to follow a negative exponential distribution. (See additional plot 12 on osf.io/6zg7a).

To obtain a representative scenario from each pool, we used the following procedure: For each pool, we went through the list of IVL scenarios obtained for that pool (see Fig 2A). The order in which we did this was, excitatory spike rates ⇒ inhibitory spike rates ⇒ number of excitatory synapses ⇒ number of inhibitory synapses, from lowest to highest values. For each scenario, a 10 second simulation was done up to 10 times, where on each iteration, presynaptic spike times and locations of active synapses were re-sampled using new random seed values. If on any of the 10 iterations, the input scenario did not satisfy the criteria for IVL, we skipped to the next IVL scenario in the list and started over. If an IVL scenario satisfied the criteria for *in vivo*-like 10 times in a row, we chose that scenario as the representative scenario for that pool, and moved on to the next pool.

Output measurements of spike amplitude, coefficient of variation of the inter-spike-interval (ISICV) and number of spikes were acquired using the Efel module in Python (see http://bluebrain.github.io/eFEL/efeature-documentation.pdf), as well as custom python code for other measurements. Specifically, measurements for subthreshold voltage mean and standard deviation, where Efel code for finding the spike-begin and spike-end indices was used to cut out all spikes from the traces in order to be left with the subthreshold portions, from which means and standard deviations were computed. All custom codes are accessible through https://github.com/FKSkinnerLab/IS3-Cell-Model. Clutter-based dimensional reordering (CBDR) plots were generated in Matlab using code obtained from Adam Taylor [39]. An illustrative example CBDR plot is shown in S1D Fig.

Input resistance, *R*_*i*_, was computed using an injected current, *I*_*inj*_, of -100 pA starting at 4.5 sec and lasting for 1 sec in the 10 sec simulation. The difference between the mean voltage during hyperpolarization and the mean voltage during baseline (0-4.5 sec, and 5.5-10 sec, with spikes cut) was divided by the current input magnitude: *R*_*i*_ = (*V*_*m*,*hyperpolarized*_ − *V*_*m*,*baseline*_)/*I*_*inj*_.

Power spectral densities (PSDs) were computed from the model’s spike train in the last 9 seconds of the simulation, using the Scipy module in Python for estimating the PSD using Welch’s method. For our bar plots, we sampled the PSD magnitude specifically at 8 Hz.

Parameter explorations were done via simulations using NEURON’s Python interface through the Neuroscience Gateway (NSG) for high-performance computing [92].

### *In vivo*-like (IVL) metric

The IS3 cell model outputs from our simulations were analyzed for the last 9 seconds of our 10 second simulations. The measurements include average subthreshold membrane potential ($\bar{V_{m}}$), standard deviation of the subthreshold membrane potential ($\sigma_{V_{m}}$), the interspike interval coefficient of variation (*ISICV*), and the average spike amplitude ($\bar{S_{A}}$). We used these four measurements in each simulation to compute our “*in vivo*-like” (IVL) metric as a conditional statement.

$$IVL\;metric=\left(\bar{V_{m}}>-66.7\;mV\right)+\left(\sigma_{V_{m}}>2.2\;mV\right)+\left(ISICV>0.8\right)-4\times (\bar{S_{A}}<40\;mV)$$ (5)

Using this equation we identified synaptic input scenarios that were *in vivo*-like (IVL metric = 3), non-*in vivo*-like (NIVL; IVL metric = 0), as well as models nearing or in depolarization block (DB; where the average spike amplitude is less than 40 mV, making the IVL metric negative). Note that the NIVL state was interpreted as not being in DB and not satisfying any of the other conditions.

To the extent of our knowledge, no *in vivo* intracellular recordings of interneurons have been performed in hippocampus or cortex. Though several studies have done juxtacellular recordings of interneurons *in vivo*, these recordings do not show the intracellular changes that are expected to occur *in vivo* versus *in vitro*. Thus, we chose the threshold values in our metric based on literature values seen in other cell types *in vivo*. For example, we know from [36] that neocortical pyramidal cells *in vivo* show elevated average subthreshold membrane potentials and subthreshold membrane potential fluctuations. We specified that the subthreshold membrane potential standard deviation should be greater than 2.2 mV. This was based on there being a 10-fold decrease in cat neocortical neuron membrane potential standard deviations *in vivo* upon application of tetrodotoxin [36]. Since the membrane potential standard deviation of the IS3 cell model without synapses was 0.22 mV (note that there is intrinsic noise applied at the soma), we could therefore expect a 10-fold increase (i.e. 2.2 mV) in an IVL state. We specified that the average subthreshold membrane potential should be greater than -66.7 mV. This was based on the change in membrane potential of mouse CA1 pyramidal cells during place field entry [47], where we would expect to see at minimum, an approximate 3 mV depolarization in the membrane potential. Alternatively, if basing this criterion on results from neocortical cells in cats from [36], we would expect a depolarization of at least 10 mV (i.e. an average membrane potential greater than -60 mV in our model). We also know that neurons *in vivo* tend to show more irregular spiking activity [35]. We specified that the interspike interval coefficient of variation (ISICV) must be greater than 0.8. This was based on a lower limit for the ISICV of neurons in middle temporal cortex of alert macaque monkeys during constant-motion stimulation [93]. Alternatively, for neurons in visual cortex and middle temporal cortex of cats and macaque monkeys, it has also been found that ISICVs greater than 0.5 could be expected *in vivo* for neurons that are spiking above 30 Hz [41].

### Excitatory and inhibitory balance metrics

To get a sense of the balance between excitation and inhibition in our explorations, we defined two excitatory-inhibitory (EI) metrics. In the first metric (Eq 6), we normalized each parameter to their maximal possible value and scaled the range such that values fall between -1 (i.e. purely inhibitory-dominant) and 1 (i.e. purely excitatory-dominant).

$$\begin{array}{c} EI\;metric\;\#1=0.5\times (\frac{N_{E_{synapses}}}{N_{max,E_{synapses}}}+\frac{f_{E}}{f_{max,E}}-\frac{N_{I_{synapses}}}{N_{max,I_{synapses}}}-\frac{f_{I}}{f_{max,I}}) \end{array}$$ (6)

Where *N* is number of synapses and *f* is spike rate for excitatory (*E*) and inhibitory (*I*) inputs. The “*max*” stands for the maximum value looked at within the parameter search (Table 4). In the second metric (Eq 7) we chose not to normalize them at all as this can unfairly penalize parameters with larger parameter ranges. Note however that while excitatory synapses have larger parameter ranges for numbers of synapses, inhibitory synapses have larger parameter ranges for presynaptic spike rates, so this possibly balances out the normalization. As a secondary E/I balance metric, we chose to simply look at the difference in cumulative spike rates across all excitatory and inhibitory synapses. Once again, any negative values represent inhibitory-dominant parameter combinations, and any positive values represent excitatory-dominant regimes.

$$\begin{array}{c} EI\;metric\;\#2=f_{E}\times N_{E_{synapses}}-f_{I}\times N_{I_{synapses}} \end{array}$$ (7)

### Isolating excitatory and inhibitory currents

To isolate the excitatory and inhibitory currents in our IS3 model cells, we ran the simulation twice, a first time where we removed inhibitory inputs, and a second time where we removed excitatory inputs. We applied a voltage clamp at the soma of the model (NEURON’s VClamp), set the leak reversal potential in all compartments to the holding potential (-70 mV for recording excitatory currents, and 0 mV for recording inhibitory currents), and removed ion channel conductances in all compartments. We also converted current traces into conductance traces according to:

$$\begin{array}{c} G_{trace}=I_{trace}/\left(V_{hold}-E_{R}\right) \end{array}$$

This protocol is similar, but not identical, to experimental protocols that measure excitatory and inhibitory currents without the use of synaptic blockers [44, 57, 58]. One notable difference is that during experiments excitatory or inhibitory currents are not removed entirely using these experimental protocols (unlike what we can easily do in our simulations by not giving input spike trains to the synapses). Rather, their activity is simply rendered ineffective by voltage clamping the cell at their supposed reversal potentials [2]. To examine how outputs might differ, we also isolated excitatory and inhibitory currents by doing the voltage clamp and without removing excitatory or inhibitory inputs directly (Fig 6). We referred to this ‘experiment-like’ one as Method 2, and the above one as Method 1.

### Estimating theta-timed synapse numbers

To determine how many excitatory and inhibitory synapses should be used for theta-timed inputs (8 Hz), we estimated the number of excitatory and inhibitory proximal and distal inputs that were necessary to elicit theta-timed spiking outputs in the *in vitro* models (i.e. when the model IS3 cell is not in an *in vivo*-like state). We did the following: (1) Approximated the number of synaptic inputs for excitatory and inhibitory synapses in either proximal (i.e. <300 *μ*m from the soma) or distal (i.e. >300 *μ*m from the soma) dendrites independently of each other. (2) For excitatory inputs, the number of inputs was increased until the IS3 cell models’ spike train PSD at 8 Hz was larger than 50 *spikes*^2^/*Hz* and there were more than 10 spikes. (3) To ensure comparability between AType+ and AType- when estimating numbers of inhibitory inputs, the magnitude of the current injection in each model was first estimated by gradually increasing the current amplitude until a spike rate of at least 35 Hz (i.e. 350 spikes in 10 seconds) was obtained in both models. Using this method, the current clamp magnitude was determined to be 27.5 pA for AType+ and 24 pA for AType-. (4) For inhibitory inputs, the number of inputs was increased until the spike train PSD at 8 Hz was larger than 80 *spikes*^2^/*Hz* and there were less than 240 spikes. The results from doing this is given in Table 5. Given these estimates, we applied 27 excitatory theta-timed synapses per layer-specific excitatory population (i.e. CA3 and ECIII, in Fig 7). Likewise, for inhibitory cell populations we applied 8 synapses per layer-specific population (i.e. bistratified, neurogliaform, OLM, IS1, and IS2, in Fig 7).

**Table 5:** Estimates of numbers of layer-specific excitatory and inhibitory theta-timed inputs required to recruit the starting IS3 cell models to spike appreciably at 8 Hz.

Model	Proximal Excitatory	Proximal Inhibitory	Distal Excitatory	Distal Inhibitory
AType+	27 Synapses	8 Synapses	27 Synapses	8 Synapses
AType-	27 Synapses	4 Synapses	18 Synapses	4 Synapses

### Further explorations done

#### Intrinsic cell noise

As described above, there are several sources of noise caused by random processes in our parameter explorations. In addition to the random choosing of synaptic locations and the random choosing of presynaptic spike times, our AType+ and AType- models have Gaussian noise current injected directly at the soma. This was originally included in our IS3 cell models in order to capture subthreshold membrane potential fluctuations that are seen experimentally [37]. To ensure that this intrinsic noise in our models was not having a major effect on our IVL state scenarios, we re-did our simulations without the intrinsic noise while keeping the random seeds for synaptic locations and presynaptic spike times fixed. As shown in Fig 4, this did not affect the consistency of our IVL scenario results.

#### Changing the number of common inputs

As described above, we estimated the number of common inputs to use for excitatory and inhibitory synapses at 9 and 4 respectively, and our examinations focused on scenarios with common inputs as they promoted *in vivo*-like states (see Results). However, these particular numbers are estimates and it is unclear whether they might strongly affect IVL states. Thus, we did further investigations using our robust, representative scenarios. We varied the number of excitatory and inhibitory common inputs from 1 (i.e. independent) to 10, and in these additional parameter explorations we kept the random seed values fixed between simulations so that only changes in the numbers of common inputs could affect the output of the simulations. The results are shown in S7 Fig. We note that there was a bit of inexactitude in these additional simulations. That is, if the total number of active synapses in the representative IVL scenario is not divisible by the number of common inputs, this leaves a remainder group of synapses of a size smaller than the number of common inputs. An example schematic of this is shown in S8 Fig. Another inexactitude with these parameter searches is that if the total number of synapses is smaller than the set number of common inputs, then increasing the number of common inputs to a larger value than the total number of available synapses would essentially change nothing (e.g. see the inhibitory common inputs axis in bottom left panel figures of S7 Fig). From this analysis, we further confirmed that the number of common excitatory inputs has a strong influence on our IVL metric (top set of plots in S7 Fig), whereas the impact of the number of common inhibitory inputs is comparatively milder. See further details in S7 Fig and caption.

## Supporting information

S1 Fig Parameter search and initial results display setup. **(A)** Schematic of common inputs versus independent inputs. **(B)** Example of randomly chosen synaptic locations along the dendritic compartments of the model, according to the parameters shown in C. Blue are proximal excitatory synapses, green are distal excitatory synapses, and red are inhibitory synapses. Note that synaptic locations can overlap on the same compartments. Apart from separating proximal and distal excitatory synaptic locations, the synaptic locations are chosen randomly. **(C)** Example raster plot of the spike times of synapses using parameter values as shown. Color scheme is the same as in B. Since this plot shows a common input scenario, note that the ‘lines’ in the raster plot are actually a series of dots (9 dots for excitatory and 4 dots for inhibitory) representing groups of synapses receiving the same (i.e., common) presynaptic spike trains. **(D)** Left: A clutter-based dimensional reordering (CBDR) plot of a parameter exploration. Example shown is for the AType+ model with common excitatory and inhibitory inputs. Excitatory input parameters are indicated by the scale bars on the y-axis and inhibitory input parameters are indicated by the scale bars on the x-axis, with parameter ranges shown in parentheses. Each pixel represents a 10 second simulation where the color of the pixel indicates the *in vivo*-like (IVL) metric score for the particular set of parameters. Using the scale bars, one can extrapolate the precise parameters of each individual pixel. Note that the height and width of the pixels are of equal size to the lengths of the smaller scale bars on the y- and x-axes. For example, going from bottom to top at an interval of the length of the larger scale bar, the excitatory spike rate increases in increments of 5 Hz. Likewise, going from bottom to top at an interval of the length of the smaller scale bar, the number of excitatory synapses increases in increments of 18 synapses, until it reaches the length of the larger scale bar, at which point it restarts the count. Similarly, the inhibitory spike rate increases in increments of 10 Hz going from left to right, and the number of inhibitory synapses increases in increments of 4 synapses. Right: Output from one of the parameter sets (a white pixel where IVL metric = 3) as indicated by the arrow. The top subplot shows the 10 second simulated voltage trace from the pixel indicated in the Left CBDR plot. Bottom subplot shows the raster plot of the presynaptic inputs where inhibitory inputs are shown in red, proximal excitatory inputs are shown in blue and distal excitatory inputs are shown in green. Note that these plots do not contain any information regarding the synaptic locations of the inputs, which are chosen randomly.
(TIFF)

S2 FigInitial results summary.CBDR plots for AType+ and AType- models and for all possible combinations of common and independent excitatory and inhibitory inputs. As described in the illustration of S1 Fig, the pixel color indicates the IVL metric score. Note that white pixels represent IVL states (IVL metric = 3), orange pixels represent NIVL states and dark pixels (i.e. red to black) represent scenarios nearing DB. The number of IVL states (white pixels) found for each AType+ independent/common condition is (from left to right): 920, 2414, 38785, 39939. Similarly for AType-: 1994, 7152, 44642, 46655. Note that there are more IVL states for common excitatory inputs and that the number of IVL states are maximized when there are common excitatory and inhibitory inputs Also note the much larger number of darker pixels for the AType- model relative to the AType+ model indicating that the AType- model enters DB much more readily than the AType+ model for the full parameter explorations. The likelihood that the AType- model is more excitable can be seen by considering the spike rates of the IS3 models that are closer to zero for the AType- models but not the AType+ models for larger excitatory spike rates and numbers of excitatory synapses. Plots of this and other separate parts of our IVL metric (Eq 5), can be seen in additional plots 1-4 on osf.io/6zg7a. Looking specifically at the subthreshold membrane potential standard deviation ($\sigma_{V_{m}}$) and interspike interval coefficient of variation (*ISICV*) metrics, we see that the larger number of IVL states with common inputs that we observe is mainly due to these characteristics having a larger parameter space that exceed their chosen thresholds to represent an IVL state. This makes sense since common excitatory or inhibitory inputs will result in a single presynaptic spike train causing larger deflections in the IS3 cell model (i.e. larger $\sigma_{V_{m}}$). Also, it is more likely that irregularly timed spiking in the IS3 cell model (i.e. larger *ISICV*) would occur since the presynaptic spike times are sampled randomly and are irregular, and excitatory input arriving at multiple synaptic locations simultaneously would increase the likelihood that the IS3 cell model could evoke a spike (see S1A Fig).(TIFF)

S3 Fig Distributions of EI metrics. Histogram distributions of EI metrics for each of the defined states (i.e. IVL, NIVL, and DB) on the same plot, and for AType+ and AType- models. Red dashed lines indicate EI metric values of zero (for both metrics) where excitation and inhibition are approximately even. Note that the IVL distributions fall within the NIVL distributions for both metrics and that the NIVL distributions tend to lean much more towards inhibitory dominant-regimes with low amounts of excitation. Looking at EI metric #2, the following can be observed: (i) In the NIVL distributions there are large peaks at zero (bottom two plots) indicating balanced excitation and inhibition. This likely represents a pool of scenarios which have zero Hz spike rates which would not be present in IVL scenarios since the requirements for an IVL state would not be satisfied. (ii) The IVL distributions have peaks just above zero, whereas the NIVL distributions do not, suggesting a subpopulation of IVL scenarios that require balanced input parameters to satisfy our requirements for an IVL state. An expanded version of only IVL states can be found in an additional plot 5 on osf.io/6zg7a.
(TIFF)

S4 Fig Distributions of EI metric #1 values for each of the 16 pools. Histogram distributions for both AType+ and AType- models. Top to bottom: IVL, NIVL, DB distributions. Consult Fig 2A for description of pool labels shown in the legend.
(TIFF)

S5 Fig Distributions of EI metric #2 values for each of the 16 pools. Histogram distributions for both AType+ and AType- models. Top to bottom: IVL, NIVL, DB distributions. Consult Fig 2A for description of pool labels shown in the legend. From the distributions in S4 Fig and S5 Fig we can once again observe that the AType- model produces a considerably larger number of DB scenarios relative to the AType+ model. We further observe that the IVL pool distributions are shifted towards more balanced metric values (i.e., EI metric values of zero), when compared to their NIVL pool counterparts. As well, the largest NIVL pool distribution (brown—HLHL), is not prominent in the IVL pool distributions plots. Likewise, the largest IVL pool distribution (grey—HHHL), is not prominent in the NIVL pool distribution plots. IVL states with large amounts of inputs (grey) tend to have inhibitory-dominant inputs and those with small amounts of inputs (blue) straddle the zero value.
(TIFF)

S6 Fig Input parameter values for the IVL representative scenario of each pool. Voltage traces and input raster plots for each representative scenario, as well as a visualization of the locations and numbers of active inputs are shown in additional plots 7-8 on osf.io/6zg7a.
(TIFF)

S7 Fig Influence of the number of common inputs. The effect of changing the number of common inputs on the IVL metric (top set of plots), subthreshold membrane potential standard deviation (middle set of plots), and the interspike interval coefficient of variation (bottom set of plots) for each representative IVL scenario from the given pool. The number of common excitatory inputs is plotted on the x-axis and inhibitory on the y-axis. (x-axis and y-axis ranges: 1 to 10 common inputs). We note that the number of common inputs shows an impact not only on the subthreshold membrane potential standard deviation (middle plots), but also on the mean subthreshold membrane potential, and the mean spike rate (see additional plot 9 on osf.io/6zg7a.). Counter-intuitively, the subthreshold membrane potential appears to mildly decrease as the number of common excitatory inputs is increased. This may be because when excitatory presynaptic spikes are more distributed (i.e. more independent) the membrane potential will be more consistently larger. On the other hand, when excitatory presynaptic spike times are correlated (i.e. more common), the increase in membrane potential caused by synaptic events will be more occasional and transient (and possibly even be partially removed from the analysis when spikes are cut from the traces), leaving the mean subthreshold membrane potential more hyperpolarized. The subthreshold membrane potential standard deviation increases as the number of excitatory (and in some cases inhibitory) common inputs is increased (middle plots). This is likely due to presynaptic spike trains causing larger deflections in the model’s membrane potential when there are larger numbers of common inputs. On the other hand, here we do not see any clear relationship between number of common inputs and the *ISICV* values, aside from occasionally regulating a border at which *ISICV* values jump from values of zero to values typically larger than 0.5 (bottom plots). The areas in the parameter space with *ISICV* values of zero appear to correspond with areas of the parameter space where there is no spiking present (see additional plot 9 on osf.io/6zg7a). Further, we observe a positive relationship between mean spike rate and number of common inputs, which demonstrates that having common correlated synaptic inputs will increase the probability of spiking. This finding is in line with work from [93] which shows that inputs need to be correlated in order to generate the irregular spiking that is often seen *in vivo*. We note that the common input numbers that we use in the main text Results (i.e. 9 common excitatory inputs, and 4 common inhibitory inputs) fall within the parameter regimes that generate IVL metric values of 3.
(TIFF)

S8 Fig Schematic illustration of inexactness. We demonstrate an example where there are 20 inhibitory inputs available, but the number of common inhibitory inputs is set to 8. This will create two groups of 8 inhibitory synapses receiving common inputs, and then a remainder group of 4 inhibitory synapses receiving common inputs. Thus our parameter exploration is inherently inexact.
(TIFF)

S9 Fig Consistency of theta recruitment. Using the LLLL representative scenario, we re-randomize synaptic locations and spike times. Bar plots at the top show the mean change in the PSD (red lines show standard deviations). Plots at the bottom show the mean *V*_*m*_ across all theta cycles in a trace (shaded areas: standard deviation; see Fig 8 for more details, and which shows excitatory and inhibitory conductances across all theta cycles). Blue traces show the baseline, and red traces show when theta-timed inputs are added.
(TIFF)
