# Supplementary figures and images for: Using computational models to predict in vivo synaptic inputs to interneuron specific 3 (IS3) cells of CA1 hippocampus that also allow their recruitment during rhythmic states
Source: PLoS One. 2019 Jan 8;14(1):e0209429. doi: 10.1371/journal.pone.0209429 (PMC6324795; doi:10.1371/journal.pone.0209429)

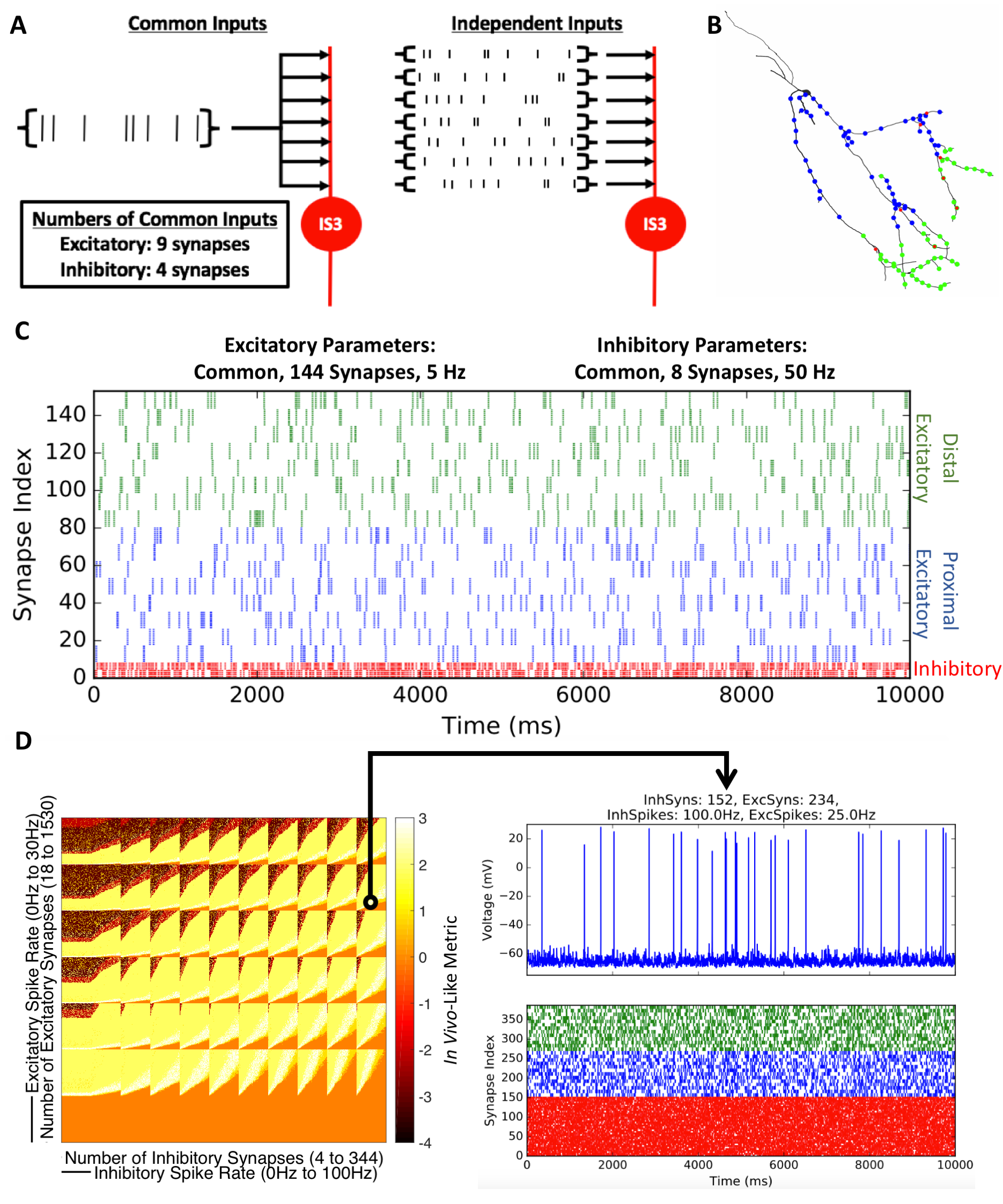

Supplement: S1 Fig — (A) Schematic of common inputs versus independent inputs. (B) Example of randomly chosen synaptic locations along the dendritic compartments of the model, according to the parameters shown in C. Blue are proximal excitatory synapses, green are distal excitatory synapses, and red are inhibitory synapses. Note that synaptic locations can overlap on the same compartments. Apart from separating proximal and distal excitatory synaptic locations, the synaptic locations are chosen randomly. (C) Example raster plot of the spike times of synapses using parameter values as shown. Color scheme is the same as in B. Since this plot shows a common input scenario, note that the ‘lines’ in the raster plot are actually a series of dots (9 dots for excitatory and 4 dots for inhibitory) representing groups of synapses receiving the same (i.e., common) presynaptic spike trains. (D) Left: A clutter-based dimensional reordering (CBDR) plot of a parameter exploration. Example shown is for the AType+ model with common excitatory and inhibitory inputs. Excitatory input parameters are indicated by the scale bars on the y-axis and inhibitory input parameters are indicated by the scale bars on the x-axis, with parameter ranges shown in parentheses. Each pixel represents a 10 second simulation where the color of the pixel indicates the in vivo-like (IVL) metric score for the particular set of parameters. Using the scale bars, one can extrapolate the precise parameters of each individual pixel. Note that the height and width of the pixels are of equal size to the lengths of the smaller scale bars on the y- and x-axes. For example, going from bottom to top at an interval of the length of the larger scale bar, the excitatory spike rate increases in increments of 5 Hz. Likewise, going from bottom to top at an interval of the length of the smaller scale bar, the number of excitatory synapses increases in increments of 18 synapses, until it reaches the length of the larger scale bar, at which point i [file pone.0209429.s001.tiff]

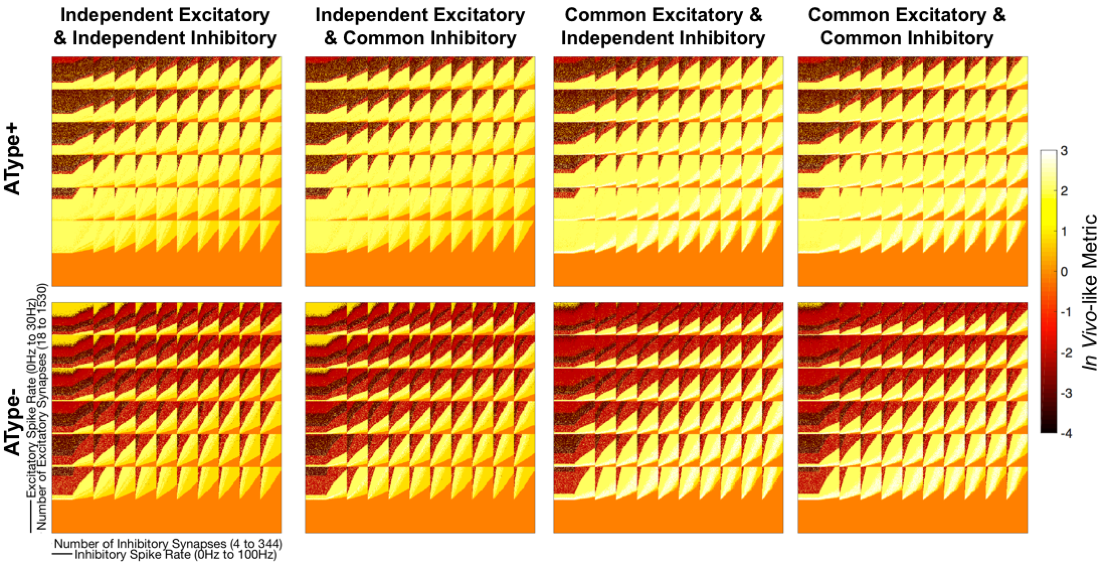

Supplement: S2 Fig — CBDR plots for AType+ and AType- models and for all possible combinations of common and independent excitatory and inhibitory inputs. As described in the illustration of S1 Fig, the pixel color indicates the IVL metric score. Note that white pixels represent IVL states (IVL metric = 3), orange pixels represent NIVL states and dark pixels (i.e. red to black) represent scenarios nearing DB. The number of IVL states (white pixels) found for each AType+ independent/common condition is (from left to right): 920, 2414, 38785, 39939. Similarly for AType-: 1994, 7152, 44642, 46655. Note that there are more IVL states for common excitatory inputs and that the number of IVL states are maximized when there are common excitatory and inhibitory inputs Also note the much larger number of darker pixels for the AType- model relative to the AType+ model indicating that the AType- model enters DB much more readily than the AType+ model for the full parameter explorations. The likelihood that the AType- model is more excitable can be seen by considering the spike rates of the IS3 models that are closer to zero for the AType- models but not the AType+ models for larger excitatory spike rates and numbers of excitatory synapses. Plots of this and other separate parts of our IVL metric (Eq 5), can be seen in additional plots 1-4 on osf.io/6zg7a. Looking specifically at the subthreshold membrane potential standard deviation (σVm) and interspike interval coefficient of variation (ISICV) metrics, we see that the larger number of IVL states with common inputs that we observe is mainly due to these characteristics having a larger parameter space that exceed their chosen thresholds to represent an IVL state. This makes sense since common excitatory or inhibitory inputs will result in a single presynaptic spike train causing larger deflections in the IS3 cell model (i.e. larger σVm). Also, it is more likely that irregularly timed spiking in the IS3 cell model (i.e. larger ISICV) would occur sinc [file pone.0209429.s002.tiff]

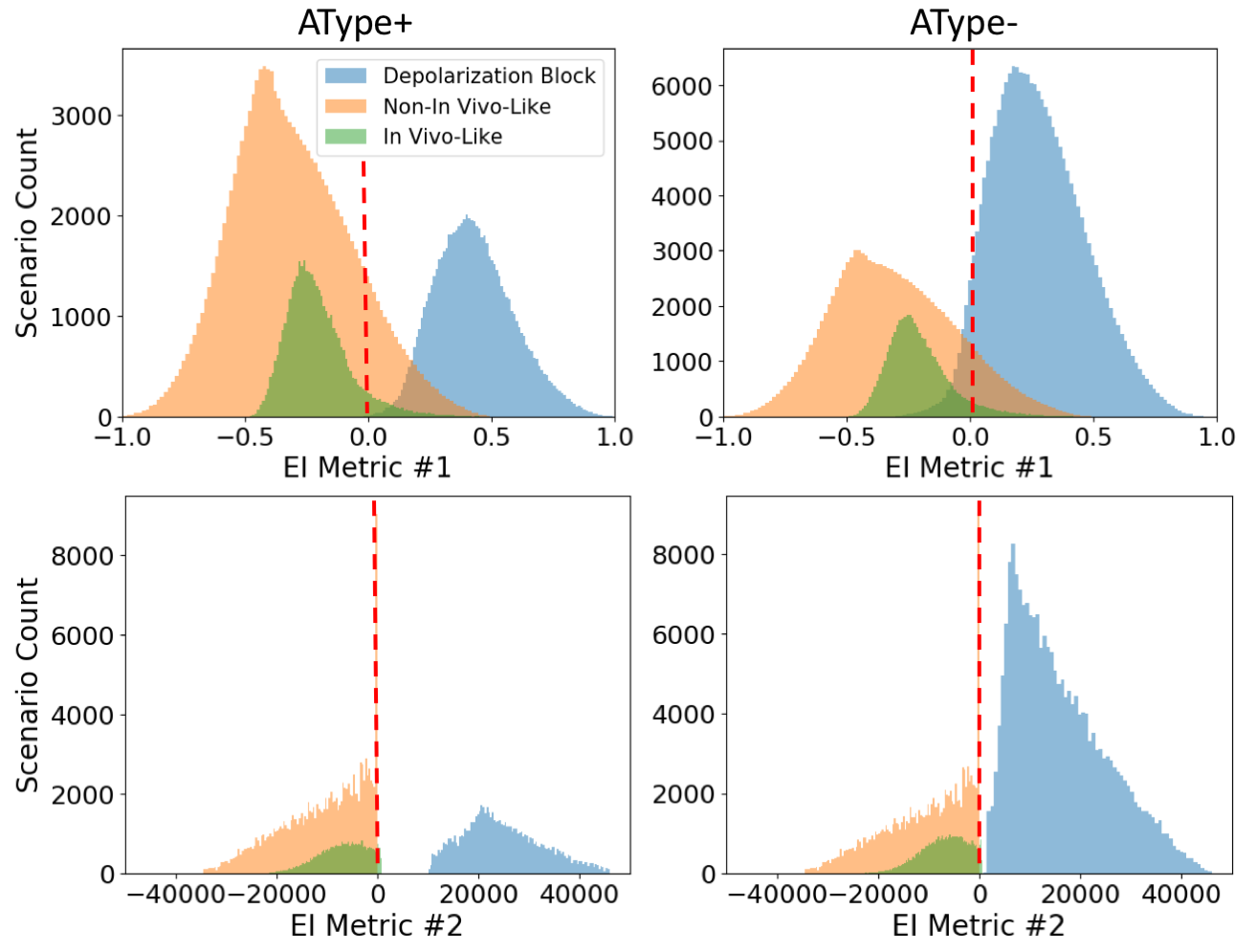

Supplement: S3 Fig — Histogram distributions of EI metrics for each of the defined states (i.e. IVL, NIVL, and DB) on the same plot, and for AType+ and AType- models. Red dashed lines indicate EI metric values of zero (for both metrics) where excitation and inhibition are approximately even. Note that the IVL distributions fall within the NIVL distributions for both metrics and that the NIVL distributions tend to lean much more towards inhibitory dominant-regimes with low amounts of excitation. Looking at EI metric #2, the following can be observed: (i) In the NIVL distributions there are large peaks at zero (bottom two plots) indicating balanced excitation and inhibition. This likely represents a pool of scenarios which have zero Hz spike rates which would not be present in IVL scenarios since the requirements for an IVL state would not be satisfied. (ii) The IVL distributions have peaks just above zero, whereas the NIVL distributions do not, suggesting a subpopulation of IVL scenarios that require balanced input parameters to satisfy our requirements for an IVL state. An expanded version of only IVL states can be found in an additional plot 5 on osf.io/6zg7a. (TIFF) [file pone.0209429.s003.tiff]

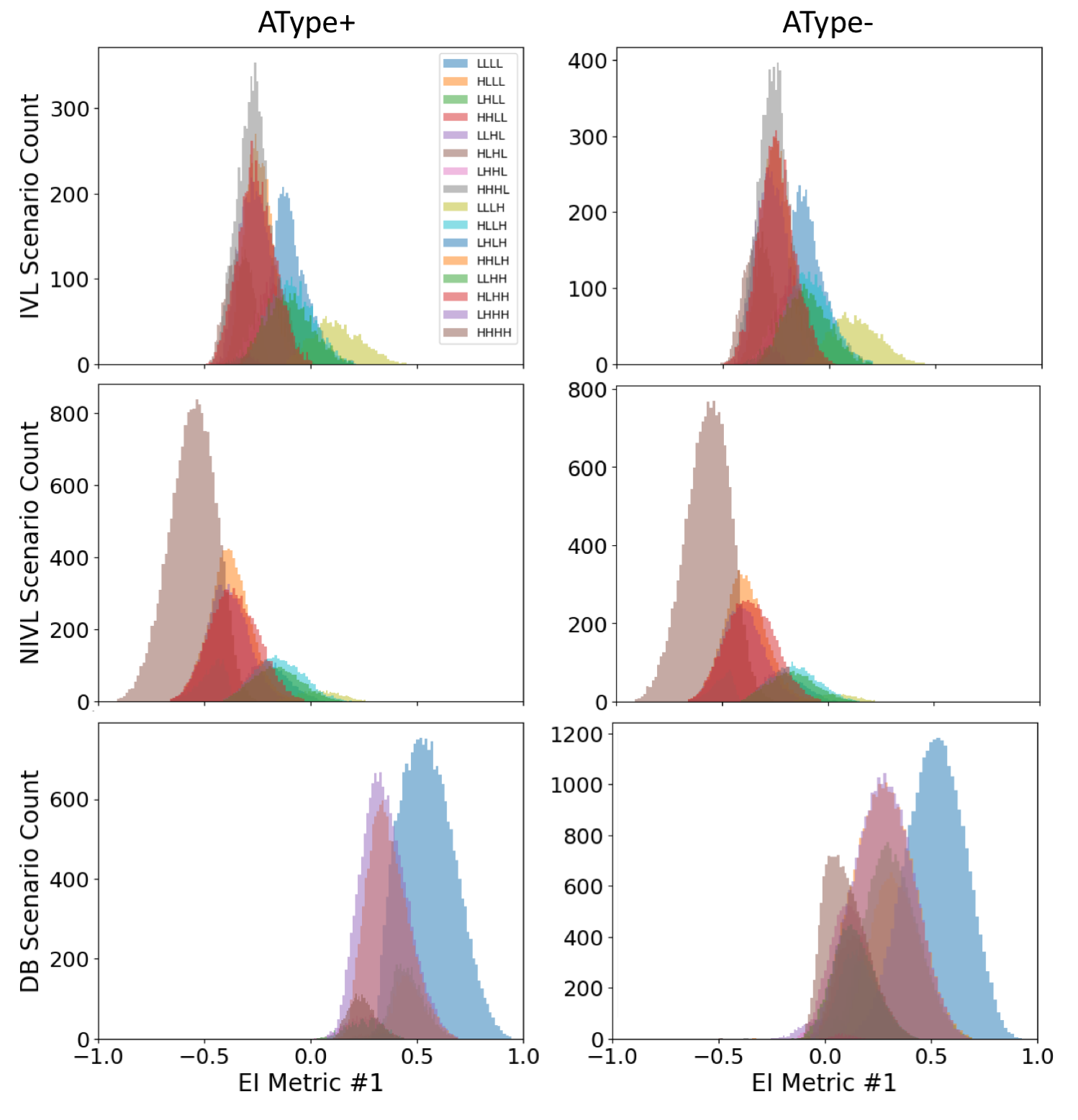

Supplement: S4 Fig — Histogram distributions for both AType+ and AType- models. Top to bottom: IVL, NIVL, DB distributions. Consult Fig 2A for description of pool labels shown in the legend. (TIFF) [file pone.0209429.s004.tiff]

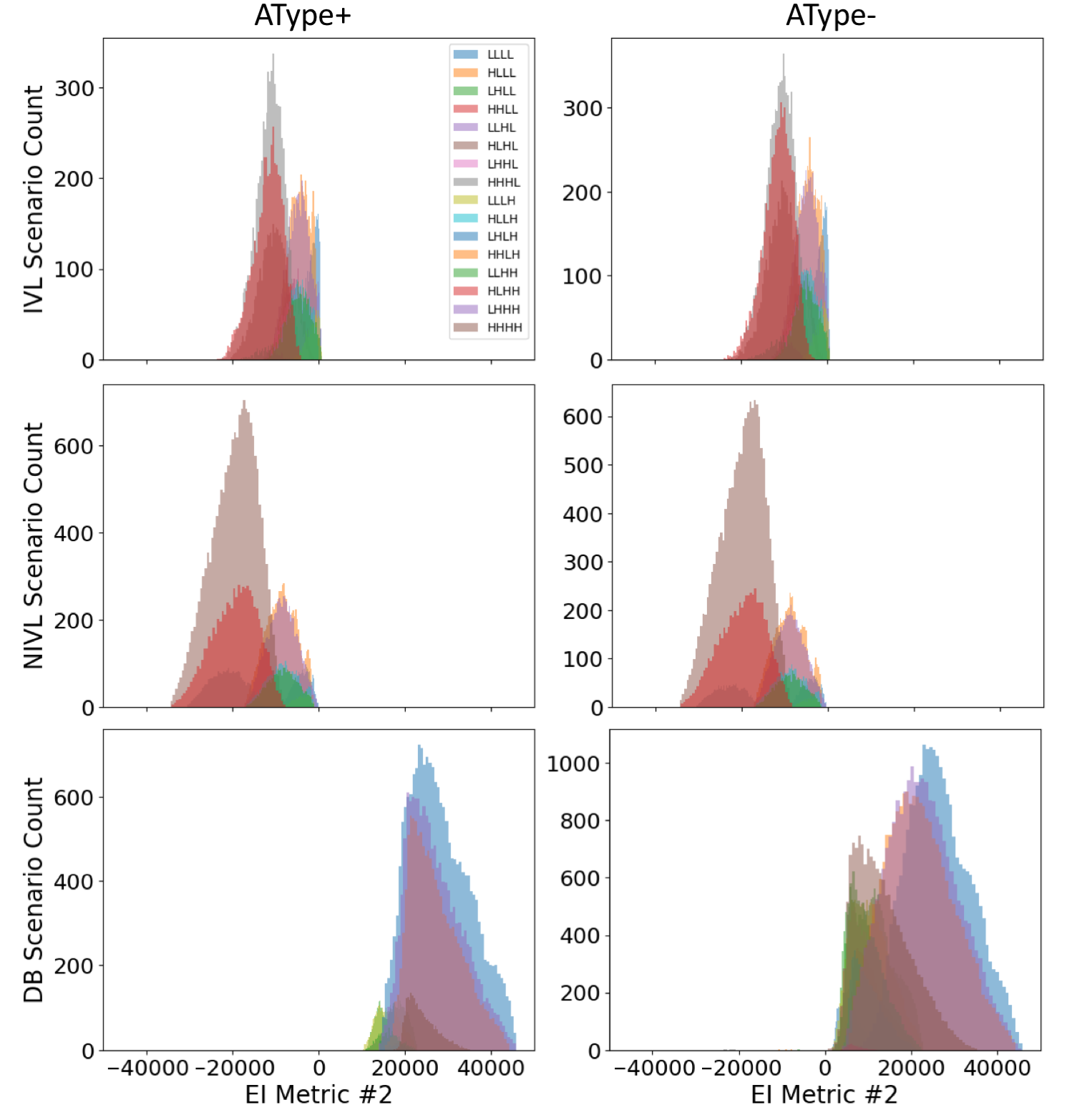

Supplement: S5 Fig — Histogram distributions for both AType+ and AType- models. Top to bottom: IVL, NIVL, DB distributions. Consult Fig 2A for description of pool labels shown in the legend. From the distributions in S4 Fig and S5 Fig we can once again observe that the AType- model produces a considerably larger number of DB scenarios relative to the AType+ model. We further observe that the IVL pool distributions are shifted towards more balanced metric values (i.e., EI metric values of zero), when compared to their NIVL pool counterparts. As well, the largest NIVL pool distribution (brown—HLHL), is not prominent in the IVL pool distributions plots. Likewise, the largest IVL pool distribution (grey—HHHL), is not prominent in the NIVL pool distribution plots. IVL states with large amounts of inputs (grey) tend to have inhibitory-dominant inputs and those with small amounts of inputs (blue) straddle the zero value. (TIFF) [file pone.0209429.s005.tiff]

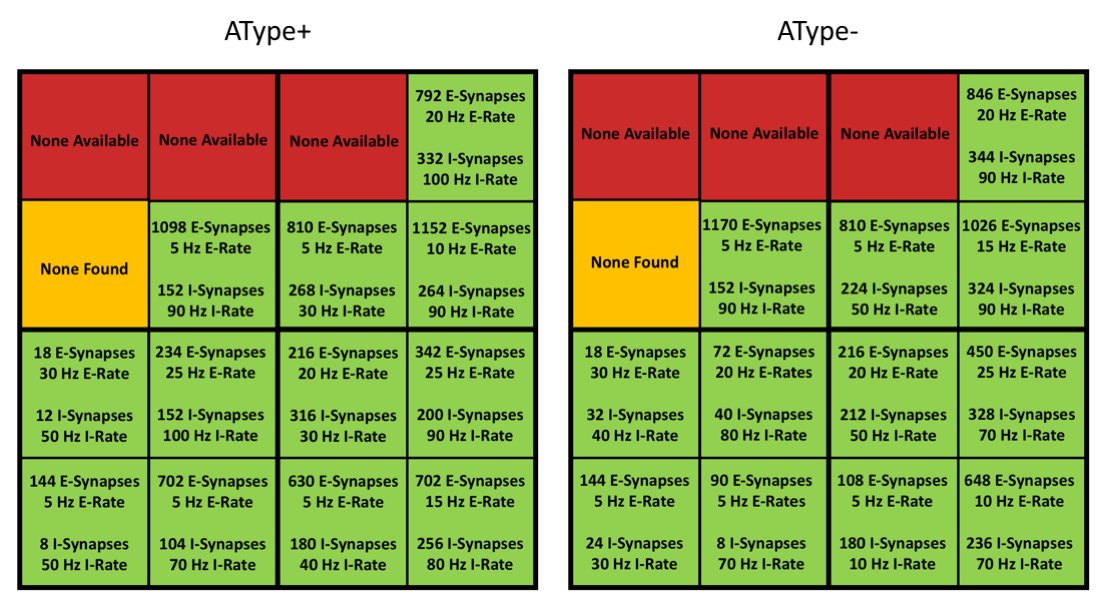

Supplement: S6 Fig — Voltage traces and input raster plots for each representative scenario, as well as a visualization of the locations and numbers of active inputs are shown in additional plots 7-8 on osf.io/6zg7a. (TIFF) [file pone.0209429.s006.tiff]

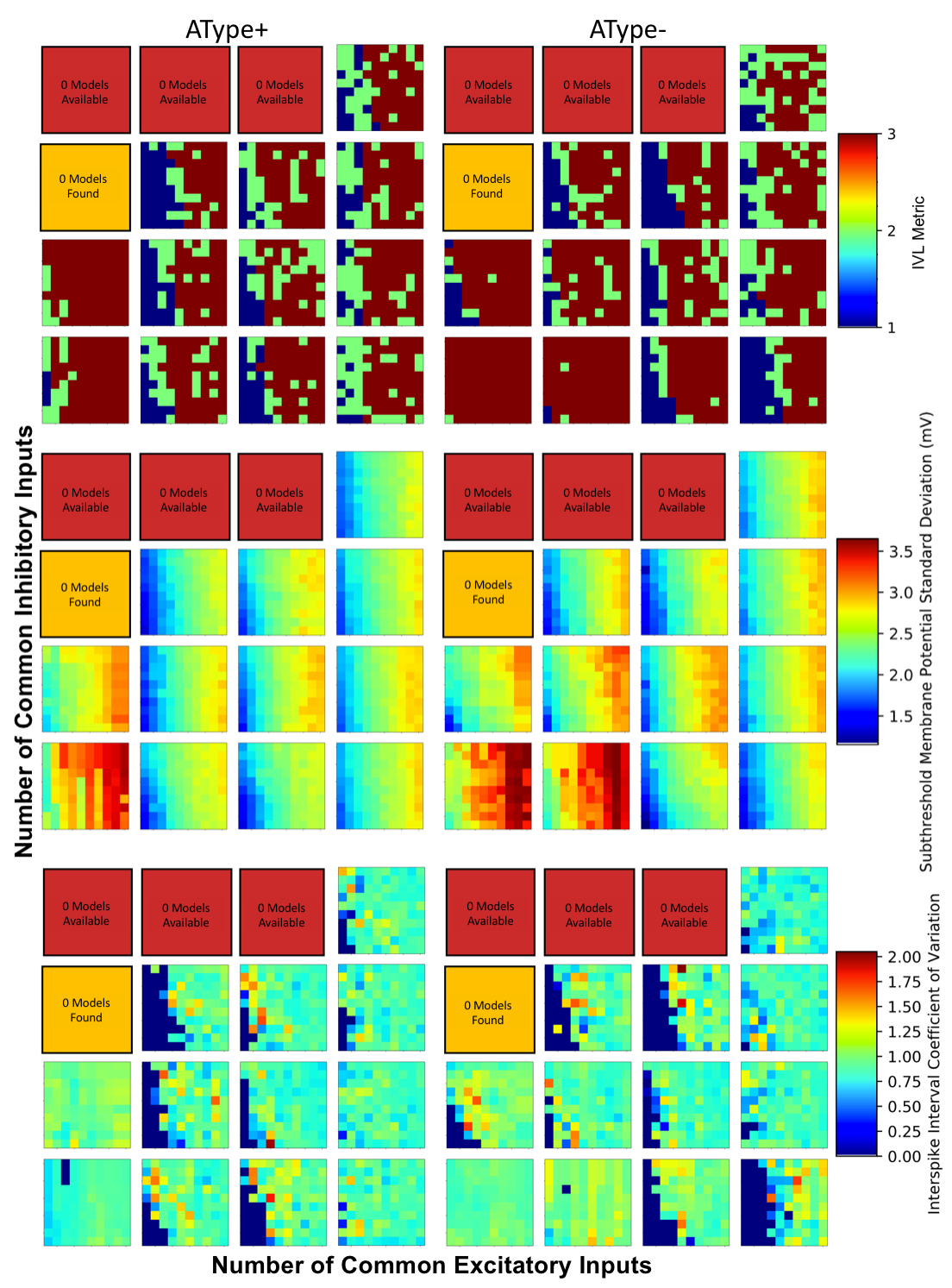

Supplement: S7 Fig — The effect of changing the number of common inputs on the IVL metric (top set of plots), subthreshold membrane potential standard deviation (middle set of plots), and the interspike interval coefficient of variation (bottom set of plots) for each representative IVL scenario from the given pool. The number of common excitatory inputs is plotted on the x-axis and inhibitory on the y-axis. (x-axis and y-axis ranges: 1 to 10 common inputs). We note that the number of common inputs shows an impact not only on the subthreshold membrane potential standard deviation (middle plots), but also on the mean subthreshold membrane potential, and the mean spike rate (see additional plot 9 on osf.io/6zg7a.). Counter-intuitively, the subthreshold membrane potential appears to mildly decrease as the number of common excitatory inputs is increased. This may be because when excitatory presynaptic spikes are more distributed (i.e. more independent) the membrane potential will be more consistently larger. On the other hand, when excitatory presynaptic spike times are correlated (i.e. more common), the increase in membrane potential caused by synaptic events will be more occasional and transient (and possibly even be partially removed from the analysis when spikes are cut from the traces), leaving the mean subthreshold membrane potential more hyperpolarized. The subthreshold membrane potential standard deviation increases as the number of excitatory (and in some cases inhibitory) common inputs is increased (middle plots). This is likely due to presynaptic spike trains causing larger deflections in the model’s membrane potential when there are larger numbers of common inputs. On the other hand, here we do not see any clear relationship between number of common inputs and the ISICV values, aside from occasionally regulating a border at which ISICV values jump from values of zero to values typically larger than 0.5 (bottom plots). The areas in the parameter space with ISICV values of zero app [file pone.0209429.s007.tiff]

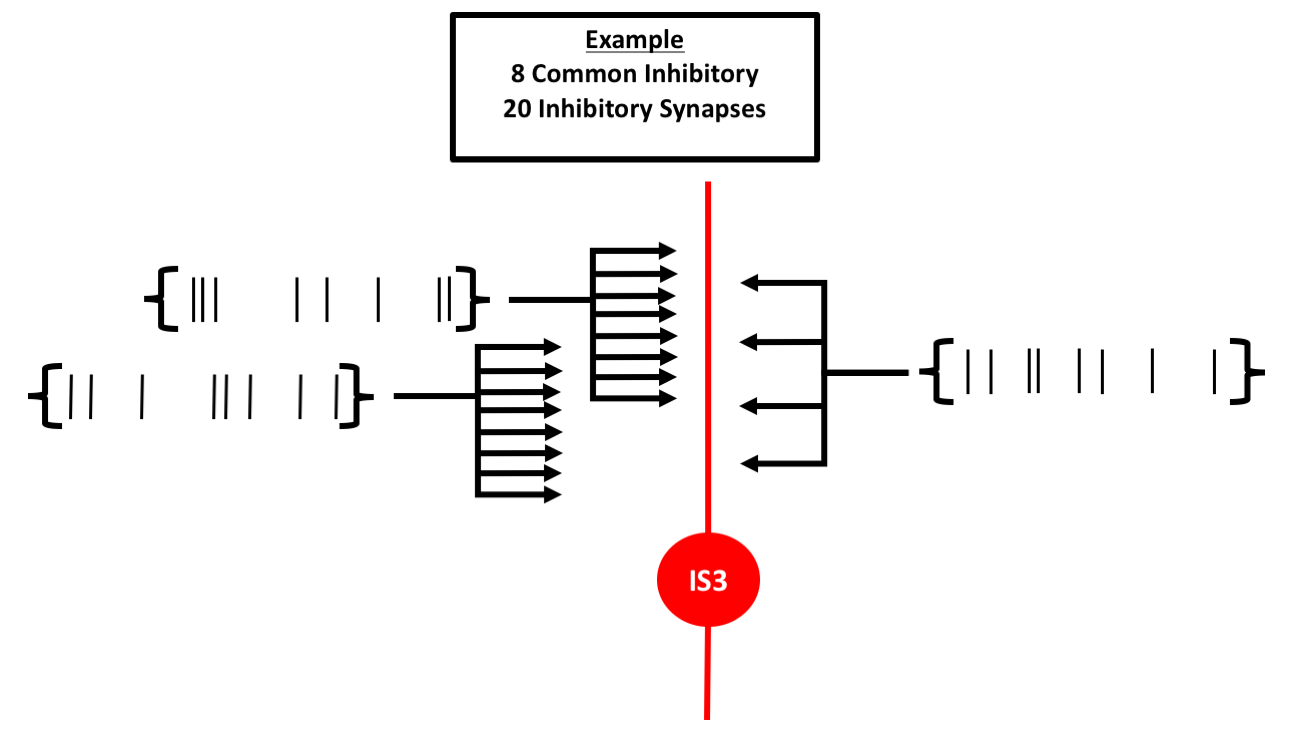

Supplement: S8 Fig — We demonstrate an example where there are 20 inhibitory inputs available, but the number of common inhibitory inputs is set to 8. This will create two groups of 8 inhibitory synapses receiving common inputs, and then a remainder group of 4 inhibitory synapses receiving common inputs. Thus our parameter exploration is inherently inexact. (TIFF) [file pone.0209429.s008.tiff]

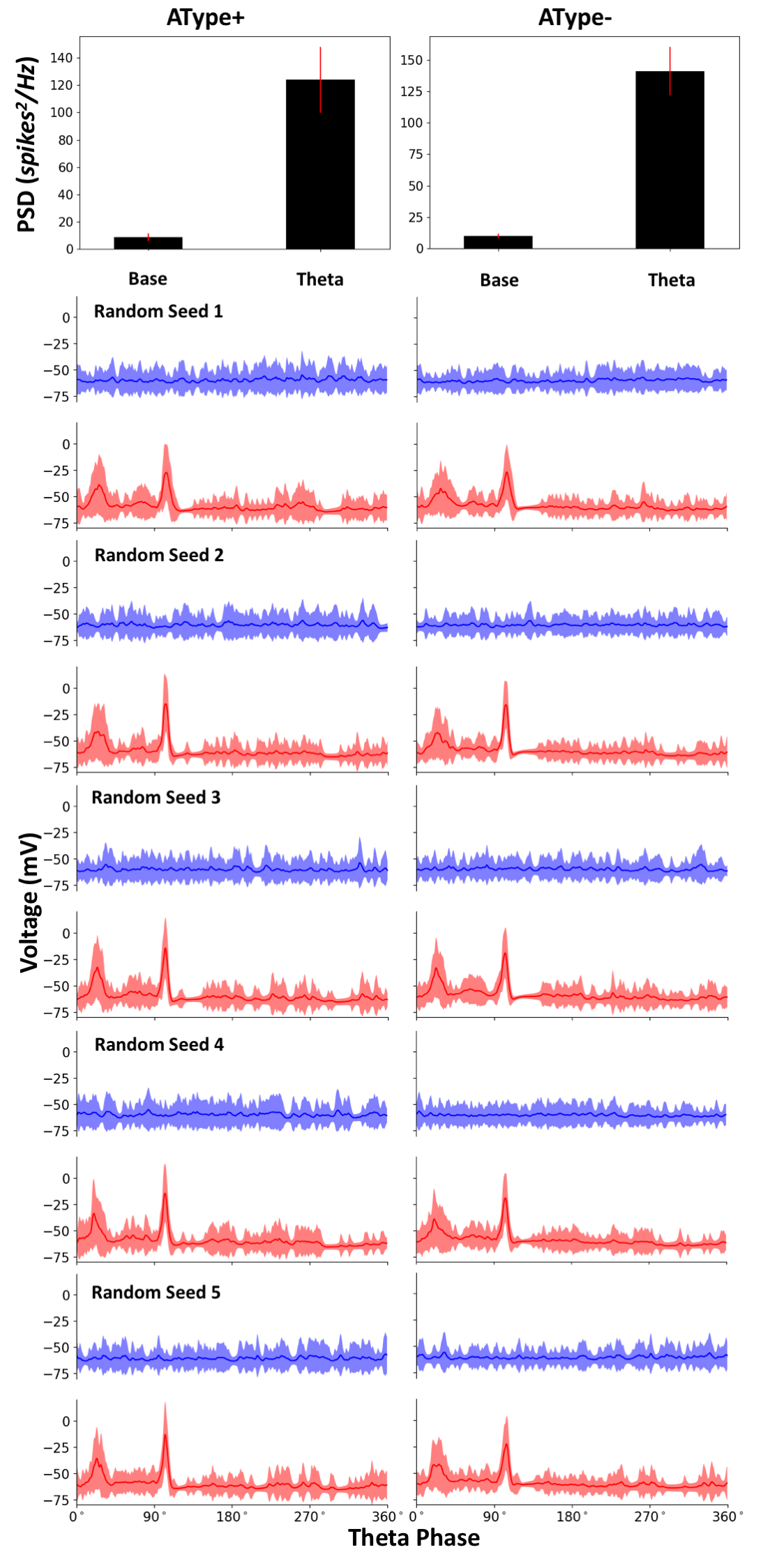

Supplement: S9 Fig — Using the LLLL representative scenario, we re-randomize synaptic locations and spike times. Bar plots at the top show the mean change in the PSD (red lines show standard deviations). Plots at the bottom show the mean Vm across all theta cycles in a trace (shaded areas: standard deviation; see Fig 8 for more details, and which shows excitatory and inhibitory conductances across all theta cycles). Blue traces show the baseline, and red traces show when theta-timed inputs are added. (TIFF) [file pone.0209429.s009.tiff]
